# Employing S(-I)/S(-II) Redox Chemistry in Quasi-1D Niobium Trisulfide-Graphene Cathodes for High-Capacity Magnesium–Lithium Hybrid Ion Batteries

**DOI:** 10.1021/acsami.5c08685

**Published:** 2025-09-25

**Authors:** Pengcheng Jing, Atsushi Inoishi, Eiichi Kobayashi, Chengcheng Zhao, Peng Ren, Isaac Abrahams, Duncan H. Gregory

**Affiliations:** † WestCHEM, School of Chemistry, Joseph Black Building, 3526 University of Glasgow, Glasgow G12 8QQ, U.K.; ‡ Institute for Materials Chemistry and Engineering, 12923 Kyushu University, Kasuga-koen 6-1, Kasuga, Fukuoka 816-8580, Japan; § 133789 Kyushu Synchrotron Light Research Center, 8-7 Yayoigaoka, Tosu, Saga 841-0005, Japan; ∥ Department of Chemistry, 4617 Queen Mary University of London, Mile End Road, London E1 4NS U.K.

**Keywords:** magnesium−lithium hybrid ion batteries, niobium trisulfide (NbS_3_), sulfur anion redox (S_2_^2−^/S^2−^) chemistry, energy storage mechanisms, high-capacity cathodes

## Abstract

Magnesium–lithium hybrid ion batteries (MLIBs) offer a compelling alternative to traditional lithium-ion batteries (LIBs) due to their enhanced safety, abundant magnesium resources, and high theoretical capacities. However, their practical deployment is constrained by the scarcity of high-capacity cathode materials. Herein, we report a quasi-one-dimensional (1D) pseudolayered niobium trisulfide (NbS_3_), incorporated with graphene, as a novel cathode that leverages disulfide (S_2_^2–^) redox chemistry to achieve remarkable electrochemical performance. The electrode exhibits excellent cycling performance, retaining high lithium/magnesium cointercalation capacities of 251 mA h g^–1^ after 150 cycles at 100 mA g^–1^ and 181 mA h g^–1^ over 1000 cycles at 1000 mA g^–1^. Mechanistic investigations using *in operando* PXRD and *ex situ* XPS/XAS reveal a two-step phase evolution during initial cycling, involving sequential sulfur anion (S_2_^2–^/S^2–^) and niobium cation (Nb^4+^/Nb^3+^) redox processes. Over extended cycling, the Nb^4+^/Nb^3+^ redox fades, leaving the S_2_^2–^/S^2–^ redox associated with a single reversible phase as the dominant contributor to the performance. The primary cause of capacity fade is attributed to the irreversible structural and redox changes related to the Nb^4+^/Nb^3+^ process, along with exfoliation-induced detachment of active material. This study provides comprehensive insights into the energy storage mechanisms of NbS_3_-based cathodes and underscores the potential of disulfide anion-containing trisulfides as promising candidates for next-generation, high-capacity magnesium-based batteries.

## Graphical abstract

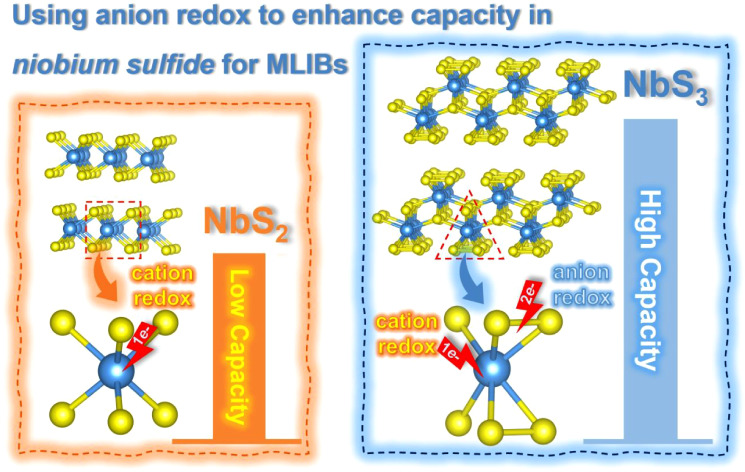

## 1 Introduction

Lithium-ion batteries (LIBs) have revolutionized energy storage, fueling the rapid growth of portable electronics and electric vehicles due to their high energy density and extended cycle life.^1^ Despite their widespread success, the increasing demand for higher energy densities, coupled with concerns over lithium scarcity and safety risks, such as flammable electrolytes and thermal runaway, necessitates the exploration of alternative battery technologies.^2^ Magnesium-ion batteries (MIBs) have emerged as a promising next-generation energy storage solution, which offer enhanced safety, abundant raw materials, high theoretical capacity, and suitable magnesium reduction potential.^3,4^ However, the divalent nature of Mg^2+^ cations introduces significant challenges, such as strong electrostatic interactions and slow diffusion kinetics, which limit the development of high-performance cathode materials.^5^

To address these challenges, magnesium–lithium hybrid ion batteries (MLIBs) have been developed by introducing lithium salts, such as LiCl, into magnesium electrolytes.^6^ This hybrid configuration combines the favorable kinetics of Li^+^ intercalation/deintercalation at the cathode with the dendrite-free Mg stripping/plating at the Mg metal anode.^6^ MLIBs provide a promising solution when MIB cathodes suffer from poor reversibility, and LIBs are constrained by the limited capacities of alternative anodes. Transition metal chalcogenides (TMCs), particularly those operating within the electrochemical stability window of many magnesium electrolytes (typically below 3.2 V), have attracted significant attention as cathode materials for MLIBs due to their superior electrical conductivity and relatively weak electrostatic interactions with intercalated ions compared to oxides.^7,8^ Layered TMCs, such as TiS_2_ and MoS_2_, are among the most extensively studied cathode materials for MLIBs, primarily due to their excellent lithium ion storage capacities in LIBs. For instance, layered TiS_2_ was reported to deliver capacities up to 220 mA h g^–1^ at a current density of 24 mA g^–1^ with stable cycling performance in MLIBs,^9^ while hydrothermally synthesized MoS_2_ nanoflowers achieved stable capacities of 210 mA h g^–1^ at 20 mA g^–1^.^10^ Metallic vanadium disulfide (VS_2_) has also been reported to exhibit reasonable cycling capacities (164 mA h g^–1^ at 50 mA g^–1^).^11^ However, conversion-type TMCs, such as CuS, NiS, and CoS_*x*_, often suffer from rapid capacity fading due to structural degradation and agglomeration of conversion products during cycling, as they lack effective ion-diffusion channels.^12–15^

Recent advancements in TMCs have highlighted the role of active S_2_^2–^ anionic redox mechanisms in enhancing electron transfer number and storage capacities.^16,17^ Pyrite-type disulfides, such as FeS_2_ and NiS_2_, offer high theoretical capacities; for instance, FeS_2_ can deliver a substantial lithiation capacity of 894 mA h g^–1^. However, their practical application is hindered by irreversible phase transitions, significant volume changes, and parasitic reactions that lead to capacity degradation. Specifically, FeS_2_ in LIBs undergoes a conversion reaction with four Li^+^ ions per FeS_2_, forming Fe and Li_2_S. Subsequent charging fails to restore the original phase, instead generating elemental sulfur that causes polysulfide shuttling.^18^ Similar behavior has also been observed for FeS_2_ in MLIBs.^15^

In contrast, quasi-one-dimensional (1D) chain-like TMCs provide structural flexibility that enables substantial ion intercalation without undergoing destructive conversion reactions. Vanadium tetrasulfide (VS_4_), featuring tetragonal-antiprismatic V coordination with eight sulfur ligands,^19^ is a notable example and has been extensively studied in LIBs, SIBs, and MIBs.^20–22^ In MLIBs, VS_4_ exhibits considerable capacities ranging from 300 mA h g^–1^ to 400 mA h g^–1^ through a cointercalation mechanism.^23–25^ Notably, aside from VS_4_, no other (quasi-)­1D TMCs have been explored in MLIBs, highlighting the importance of investigating analogous electrode materials.

In this study, we report for the first time the application of a quasi-1D pseudolayered niobium trisulfide (NbS_3_), incorporated with graphene, as a novel cathode material for MLIBs. NbS_3_ features a chain-like structure composed of Nb^4+^ cations coordinated with S_2_^2–^ and S^2–^ anions, providing abundant ion storage sites enabled by reversible S_2_^2–^/S^2–^ redox reactions. The electrode demonstrates promising cycling performance, retaining high lithium/magnesium cointercalation capacities of *ca*. 251 mA h g^–1^ after 150 cycles at 100 mA g^–1^ and *ca.* 181 mA h g^–1^ after 1000 cycles at 1000 mA g^–1^. Mechanistic investigations, including *in operando* powder X-ray diffraction (PXRD), *ex situ* X-ray photoelectron spectroscopy (XPS), and X-ray absorption spectroscopy (XAS), elucidate phase/structural transitions and a sulfur anion-based (S_2_^2–^/S^2–^) redox mechanism, which underpins the substantial lithium/magnesium ion cointercalation during extended cycling. This work highlights the potential of S_2_^2–^/S^2–^ redox chemistry in intercalation trisulfide compounds as a promising strategy for the development of next-generation cathode materials for MLIBs.

## 2 Experimental Section

All experiments described herein were conducted at room temperature (RT) unless explicitly stated otherwise.

### 2.1 Physical Vapor Transport (PVT) Synthesis of NbS_3_

NbS_3_ was synthesized using a PVT method. In a typical procedure, a slightly overstoichiometric molar ratio of niobium (99.8%, 325 mesh, Aldrich; 0.464 g) and sulfur (Sigma-Aldrich, 99.98%; 0.490 g) powders (1:3.06) were thoroughly mixed in an agate mortar in a high-purity argon-filled glovebox. The powder mixture was sealed in a quartz tube under a static vacuum of *ca*. 10^–4^ mBar. The sealed tube was placed horizontally in a box furnace (Brother Furnace, BR-12N-5) and heated using the following profile: ramped at 200 °C h^–1^ to 115 °C and dwelled for 3 h, then heated at 200 °C h^–1^ to 550 °C for 82 h.^26^ Following the reaction, the tube was allowed to cool naturally to RT inside the furnace over a period of 4 h. The quartz tube was opened in the glovebox using a Mo glass cutter, and the resulting product was collected, ground into finer particles, and stored in the glovebox for further use.

### 2.2 Preparation of NbS_3_@Graphene Composite

The NbS_3_@15 wt % graphene composite (NbS_3_G15) was prepared by low-energy mechanical milling, which offers a simple, scalable, and solvent-free approach to achieve homogeneous mixing. This process also helps reduce particle aggregation of the as-synthesized NbS_3_ without altering its crystal structure. A mixture of 0.425 g as-synthesized NbS_3_ and 0.075 g graphene was loaded into a 50 mL stainless steel milling jar along with five stainless steel balls (each 4 g; ball-to-powder mass ratio of 40:1) under an argon atmosphere. The sealed jar was then subjected to ball milling at 400 rpm for 3 h, employing alternating 2 min forward and reverse rotations separated by 2 min pauses. The resulting black powder was collected and stored in an argon-filled glovebox for subsequent use. For the graphene-free NbS_3_ electrode, NbS_3_ and conductive carbon were premixed using the same milling method.

#### 2.2.1 Material Characterization

The as-prepared and cycled samples were systematically characterized using powder X-ray diffraction (PXRD), Raman spectroscopy, thermogravimetric analysis (TGA), X-ray photoelectron spectroscopy (XPS), X-ray absorption spectroscopy (XAS), scanning electron microscopy (SEM), and transmission electron microscopy (TEM).

Typically, PXRD patterns were collected in Bragg–Brentano geometry (flat plate, reflection) over a 2θ range of 5° – 70°, with a step size of 0.0175° and a scan rate of 0.083° s^–1^ using a Rigaku MiniFlex diffractometer (unmonochromated Cu K_α_, 40 kV, 40 mA). *In operando* PXRD was conducted using a PANalytical Empyrean diffractometer with the same X-ray source, over a range of 5° – 70°, a step size of 0.0263°, and a scan rate of 0.074 ° s^–1^, using a bespoke CR2032 coin cell with a Kapton window (8 mm diameter). Raman spectra were recorded using a LabRAM HR spectrometer equipped with a 532 nm green laser. Powdered samples were pressed onto glass slides and measured in air. TGA was performed on a TA Instruments SDT Q600 instrument by heating samples from 25 to 600 °C (or 1000 °C) at a rate of 10 °C min^–1^ under a flow of 2% O_2_/98% Ar atmosphere (100 mL min^–1^). XPS measurements were carried out using Thermo Scientific Nexsa and Jeol Jsps-9010MC/IV spectrometers with Al Kα and Mg Kα X-ray sources, respectively. High-resolution spectra were fitted using dual Gaussian–Lorentzian functions according to the method of Conny and Powell.^27,28^ For *ex situ* XAS analysis, two types of samples were prepared: uncycled NbS_3_G15 powder and cycled NbS_3_G15 electrodes (3 mm × 5 mm; on carbon paper). Each sample was adhered onto carbon conductive tape, transferred into a custom-made transfer vessel within an argon-filled glovebox, transported to the beamline for measurements without air exposure. XAS measurements at the Nb L-edges (2367–2473 eV) and S K-edges (2460–2490 eV) were conducted at the BL2A beamline of the UVSOR Synchrotron Facility (Institute for Molecular Science, Japan). Spectra were collected in total electron yield mode and calibrated using the S K-edge spectrum of Li_2_S. The data provided insights into the oxidation states and coordination environments of niobium and sulfur.

SEM-EDS analysis was conducted using a Tescan Clara microscope equipped with an Oxford Instruments UltimMax 65 energy dispersive X-ray spectrometer operated at 15 kV. Powder samples were prepared by dispersing them onto conductive carbon tape in air, followed by coating with a thin (*ca.* 10 nm) gold layer via plasma sputtering. Electrochemically cycled electrodes were prepared in the glovebox without gold coating, sealed in 14 mL vials, and promptly transferred to the SEM chamber, limiting air exposure to less than 10 s. TEM imaging was performed on a FEI Tecnai G2 F30 Microscope operated at 300 kV. For sample preparation, approximately 1 mg of as-synthesized NbS_3_G15 powder was dispersed in 1 mL of absolute ethanol using ultrasonication for 5 min. A drop of the resulting suspension was deposited onto an ultrathin carbon-coated Cu TEM grid and allowed to dry in air for 1 h.

#### 2.2.2 Electrochemical Measurements

The electrochemical performance of NbS_3_G15 and NbS_3_ electrodes was evaluated using CR2032-type coin cells. The electrodes were prepared via a slurry-coating method. Slurries were made by blending 0.070 g of active material, 0.020 g of conductive carbon (carbon black, 99%, Alfa Aesar), and 0.010 g of poly­(vinylidene fluoride) (PVDF, 98%, M_*n*_ ≈ 534,000, Sigma-Aldrich) binder in approximately 0.45 mL of *N*-methyl pyrrolidone (NMP, anhydrous, 99.5%, Sigma-Aldrich). The resulting slurry was coated onto carbon paper discs (diameter 12 mm) and dried overnight in a vacuum oven prior to cell assembly. Magnesium metal foil discs (0.2 mm thickness, 15 mm diameter, 99.5%, Huabei Magnesium Processing Plant) served as anodes. Glass fiber discs (Whatman, GF/D, 16 mm diameter) were used as separators. Molybdenum foil discs (16 mm diameter, precleaned with ethanol) were placed between the electrode current collectors and cell case pieces to prevent electrolyte corrosion. The mass loading of cathode material was in the range of 1.14–2.14 mg per electrode (equivalent to 1.00–1.91 mg cm^–2^).

The 0.4 M “all-phenyl complex” (APC) electrolyte was prepared by the dropwise addition of 2.0 mL phenyl magnesium chloride solution (PhMgCl, 2.0 M in tetrahydrofuran (THF), Sigma-Aldrich) to 3.0 mL THF (≥99.9%, anhydrous, inhibitor-free, Sigma-Aldrich) solution of AlCl_3_ (0.267 g) under magnetic stirring. The 1 M LiCl-APC hybrid ion electrolyte was prepared by dissolving 0.085 g of LiCl (≥99.9%, Aldrich) in 2.0 mL of 0.4 M APC solution and stirring overnight inside an argon-filled glovebox. For comparison, LIB cells were assembled using 1.0 M LiPF_6_ in EC/DEC = 50/50 (v/v) (battery grade, Sigma-Aldrich) as the electrolyte. Approximately 90 μL of electrolyte was added to each coin cell.

Galvanostatic (dis)­charge and galvanostatic intermittent titration technique (GITT) tests were performed using a Land CT2001A battery tester in the voltage range of 0.1–2.2 V. For GITT, the cell was discharged/charged at 50 mA g^–1^ for 600 s, followed by a relaxation period of 1200 s under open-circuit conditions. This process was repeated until the lower or upper voltage limit (0.1 or 2.2 V) was reached. Details of the GITT data analysis for diffusivity calculations are provided in Figure S16 and eq S4. Cyclic voltammetry (CV) was conducted over the same voltage range (0.1–2.2 V) at scan rates of 0.1–0.8 mV s^–1^, beginning with the cathodic sweep. Electrochemical impedance spectroscopy (EIS) were carried out over a frequency range of 100 kHz to 0.01 Hz with a 10 mV amplitude. Both CV and EIS measurements were performed using a PalmSens4 potentiostat.

## 3 Results and Discussion

NbS_3_ was synthesized via a simple physical vapor transport (PVT) method, as detailed in the Experimental section. The PXRD patterns of NbS_3_ with 15 wt % of graphene (NbS_3_G15) and pure NbS_3_ are shown in Figure 1a. Both patterns align with the triclinic NbS_3_ phase (PDF-71–0468), which crystallizes in the space group of *p*-1. The disproportionately high intensity of the (001) reflection is attributed to a preferred orientation in the 00*l* direction. Interestingly, diffraction peaks corresponding to (*h*0*l*) planes, such as (10–1) and (101), are absent, consistent with previous observations in pseudolayered NbS_3_ and layered disulfides (e.g., MoS_2_ and WS_2_).^26,29,30^ This phenomenon is attributed to stacking disorder in the pseudolayers, caused by lateral shift of individual layers along the *a*-axis. Figure 1b-d demonstrate the pseudolayered crystal structure of triclinic NbS_3_. In this structure, [NbS_6_] trigonal prisms (highlighted in light violet) connect along the *b*-axis via polar covalent S–Nb–S bonds to form infinite 1D chains (light pink bands). These chains are aligned along the *a*-axis and are held together by weaker Nb–S interchain covalent interactions. This arrangement gives rise to pseudolayers (light gray bands) that stack through weak van der Waals (vdWs) forces within the *ab* plane. The Raman spectra of NbS_3_G15 and pure NbS_3_ are shown in Figure 1e, with signals closely matching those reported in the literature.^31^ Specifically, the peak at *ca*. 150 cm^–1^ is attributed to a T_*y*_^′^ “rigid-chain” motion caused by lattice compression along the chain,^31^ while the shoulder peak at *ca*. 159 cm^–1^ corresponds to Nb–Nb stretching. The prominent signal at *ca*. 193 cm^–1^ arises from Nb–S_2_^2–^ vibrations, and the medium-to-low frequency peak at *ca*. 260 cm^–1^ suggests the presence of symmetrical Nb–S “rigid-sublattice” motions along the *b*-axis due to the atomic displacements. The two faint peaks at *ca*. 298 cm^–1^ and *ca*. 319 cm^–1^ are attributed to Nb–S intrachain vibrations, while the pronounced bands ranging from *ca*. 337 cm^–1^ to *ca*. 348 cm^–1^ correspond to Nb–S^2^− vibrations, which remain unassigned between interchain and intrachain origins. The two neighboring peaks at *ca*. 377 cm^–1^ and 387 cm^–1^ are ascribed to interchain Nb–S^2^− vibrations, while the high-frequency peak at *ca*. 570 cm^–1^ is associated with S–S bond vibrations in disulfide (S_2_^2–^) bridges. The inset highlights the graphene D band (*ca*. 1340 cm^–1^) and G band (*ca*. 1594 cm^–1^) in NbS_3_G15.^32^ XPS analysis was performed to investigate the surface chemical states of Nb and S in NbS_3_G15. The high-resolution Nb 3d spectrum (Figure 1f) reveals four distinct peaks. The doublet peaks at *ca.* 203.9 eV (3d_5/2_) and 206.6 eV (3d_3/2_) correspond to Nb^4+^ in NbS_3_.^33,34^ In contrast, the peaks at *ca*. 207.1 and 209.8 eV are assigned to Nb^5+^ in Nb_2_O_5_, indicating surface oxidation.^35^ Turning to the high-resolution S 2p spectrum (Figure 1g), two pairs of doublet peaks are observed. The peaks at *ca*. 161.2 eV (2p_3/2_) and 162.3 eV (2p_1/2_) correspond to S^2–^ species, while those at *ca*. 162.4 and 163.6 eV are attributed to S_2_^2–^ species in NbS_3_.^36–38^

**Figure 1:**
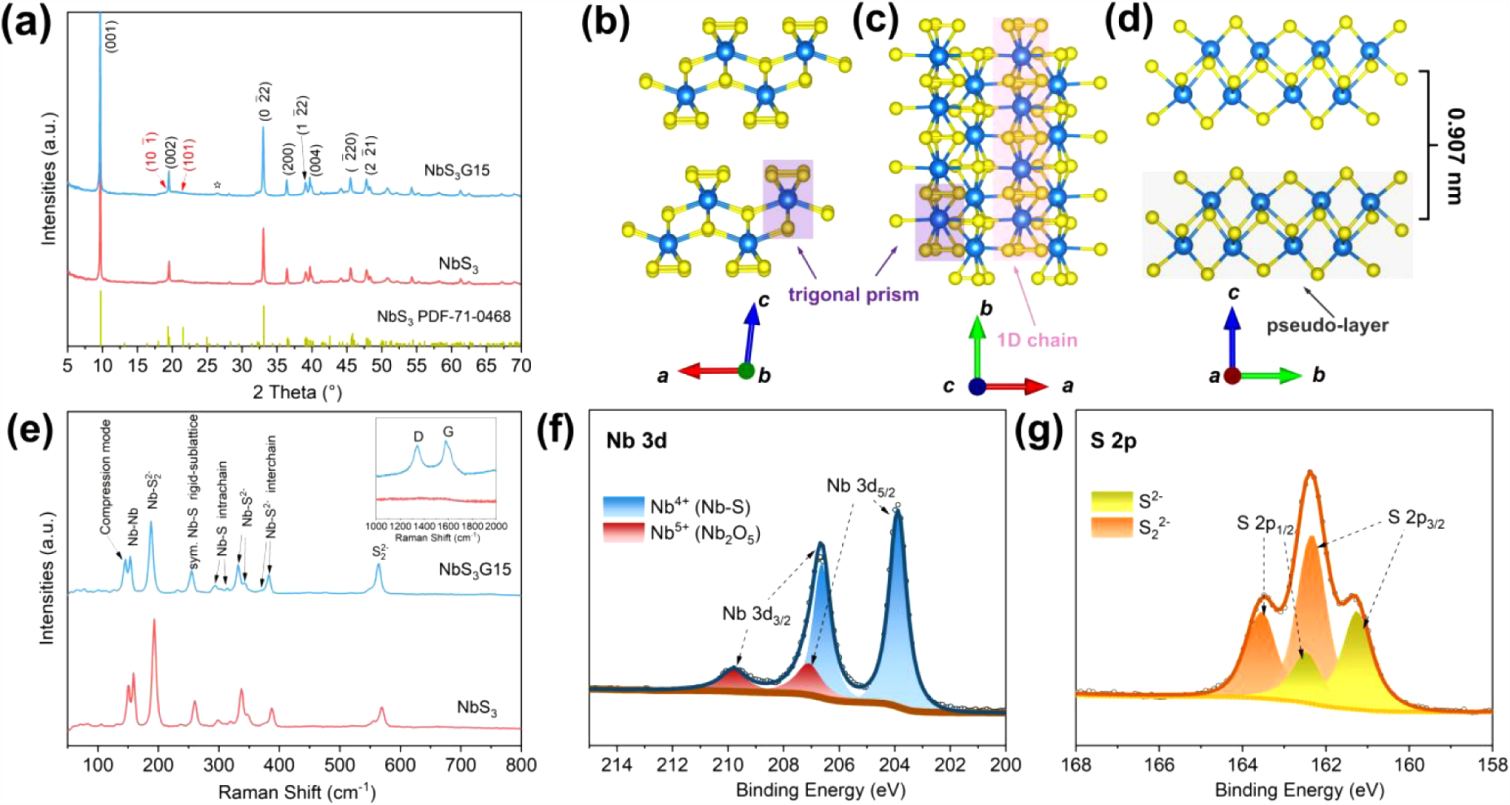
(a) PXRD patterns of NbS_3_G15 and NbS_3_. The peak marked with a star indicates the characteristic diffraction of graphene in the NbS_3_G15 sample. Crystal structure of NbS_3_ projected onto the (b) *ac*, (c) *ab*, and (d) *bc* planes, as visualized by Vesta software.^39^ The following components are highlighted: [NbS_6_] trigonal prisms (light purple shadows); 1D S–Nb–S chains (light pink shadow), NbS_3_ pseudolayers (light gray shadow), and interlayer spacing (*ca*. 0.907 nm). (e) Raman spectra of NbS_3_G15 and NbS_3_. High-resolution XPS spectra of: (f) Nb 3d and (g) S 2p transitions in NbS_3_G15.

SEM-EDS were initially employed to investigate the morphology and composition of the NbS_3_G15 and NbS_3_ samples. The low- and high-magnification SEM images in Figures 2a,b show that the NbS_3_ component in NbS_3_G15 exists as micro/nano rods, with widths spanning several hundred nanometers and lengths extending to several micrometers. Notably, the surface of these rods appears rough and is likely decorated with smaller graphene pieces, which contrasts with the smoother surface observed in the pure NbS_3_ sample (Figure S1). This suggests a relatively uniform mixing of NbS_3_ and graphene during ball milling. Figure 2c shows the SEM image and corresponding EDS elemental maps of Nb, S, and C for the NbS_3_G15 sample. The maps confirm a uniform distribution of Nb and S, while carbon appears to be dispersed on or among the NbS_3_ particles. However, a significant portion of the carbon signal, particularly in areas not overlapping with Nb and S, is attributed to the carbon tab used for sample mounting. This is consistent with the EDS spectrum (Figure S2), which shows a prominent carbon peak. The S/Nb molar ratio derived from the EDS spectrum is 2.78 ± 0.05 approximating the stoichiometry of NbS_3_. The slight sulfur deficiency may be attributed to surface oxidation or hydrolysis, evidenced by the presence of oxygen in the spectrum and the emission of sulfur-containing gas upon air exposure. While EDS provides semiquantitative results, its accuracy can be affected by surface irregularities, as traditionally protocols involve polished samples. To obtain a more precise stoichiometric ratio, TGA analysis was performed on the NbS_3_ sample in an oxidizing O_2_/Ar atmosphere. The results confirm complete conversion to niobium oxide (Nb_2_O_5_), yielding an S/Nb molar ratio of 2.89 ± 0.02 (Figure S3). For the NbS_3_G15 composite, TGA under identical conditions confirms a graphene content of 15.8 ± 0.3 wt %, consistent with the target carbon proportion (15 wt %) in the composite (Figure S4).

**Figure 2:**
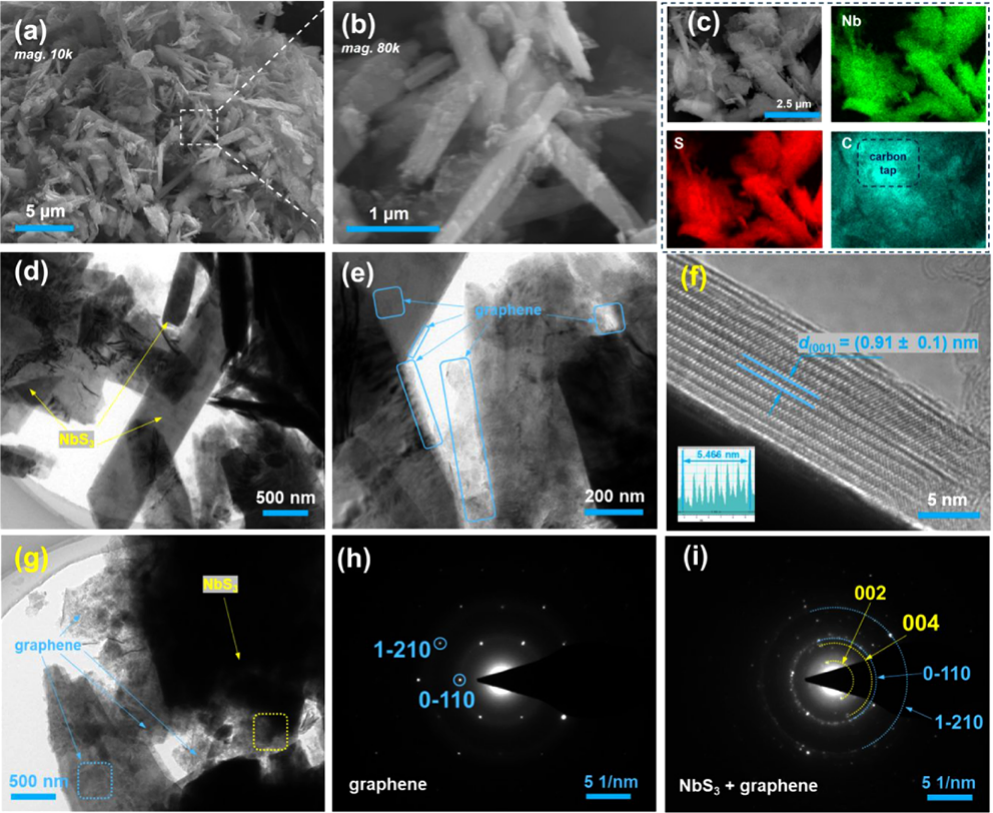
Electron microscopic characterization of NbS_3_G15: (a) Low-magnification (*10k*) and (b) higher-magnification (*80k*) SEM images. (c) SEM image with corresponding EDS elemental maps for Nb, S, and C, respectively. (d) and (e) TEM images, (f) HRTEM image with an embedded graph measuring the lattice fringe, and (g) TEM image and corresponding SAED patterns acquired in (h) blue dashed line area and (i) yellow dashed line area.

TEM analysis further reveals the microstructure of NbS_3_G15. As shown in Figure 2d, micro- and nano rods of NbS_3_ (indicated by yellow arrows) are dispersed and stacked, with widths ranging from 300 to 600 nm and lengths between 1 and 5 μm. Higher-magnification TEM imaging (Figure 2e) shows small graphene fragments (highlighted by blue boxes) either coated onto the surfaces of the rods or dispersed among them, indicating intimate mixing between NbS_3_ and graphene and the successful formation of a composite structure. The HRTEM image in Figure 2f, supported by the inserted measurement graph generated using DigitalMicrograph software,^40^ reveals distinct lattice fringes with an interlayer spacing of 0.91 (1) nm, corresponding to the (001) crystal plane of NbS_3_. The single crystal SAED pattern in Figure 2h, obtained from the region marked by blue dashed lines in Figure 2g, is attributed to multilayer graphene dispersed among the NbS_3_ particles. The polycrystalline SAED pattern in Figure 2i, acquired from the yellow-circled region in Figure 2g, displays diffraction rings corresponding to the (002) and (004) planes of NbS_3_, as well as the (0–110) and (1–210) planes of graphene.

A series of electrochemical experiments were conducted to evaluate the electrochemical performance of NbS_3_G15 and NbS_3_ as cathode materials for MLIBs. Cyclic voltammetry (CV) measurements were first carried out to examine the effect of Li addition on the electrochemical behavior of the NbS_3_G15 electrode. CV curves were recorded in both a pure Mg ion electrolyte (APC, 0.4 M 2PhMgCl-AlCl_3_ in tetrahydrofuran) and a hybrid Mg–Li ion electrolyte (LiAPC, 1.0 M LiCl + 0.4 M APC) at a scan rate of 0.3 mV s^–1^ over a voltage range of 0.1–2.2 V. In the APC electrolyte (Figure S5a), the first scan reveals an irreversible and weak cathodic/anodic redox couple at *ca*. 0.6 and 0.2 V (cathodic)/1.9 V (anodic). Subsequent cycles show a gradually fading redox couple at *ca*. 1.0 V/1.7 V with minimal current response, indicating negligible magnesium ion storage. In contrast, the LiAPC electrolyte induces pronounced redox activity, as shown in Figure 3a. In the first cathodic scan, two prominent peaks are observed at *ca*. 0.67 V (from 0.89 to 0.56 V) and *ca*. 0.48 V (from 0.56 to 0.34 V), which are attributed to the sequential reduction of S_2_^2–^ to S^2–^ and Nb^4+^ to Nb^3+^, respectively. This assignment is consistent with our mechanistic studies in the following sections and the electrochemical lithiation behavior reported for NbS_3_ and other tricalcogenides in LIBs.^41–43^ During the first anodic sweep, three peaks appear at *ca*. 0.97, 1.49, and 1.86 V. The first corresponds to the oxidation of Nb^3+^ to Nb^4+^, while the latter two are associated with the reformation of S_2_^2–^. These peak positions and intensities differ markedly from those observed in the APC electrolyte, suggesting that Li^+^ ions, rather than magnesium ions, are likely the dominant species involved in the (de)­insertion processes. In the subsequent cycles, the cathodic and anodic peaks of the high-voltage redox couple (S_2_^2–^/S^2–^) shift toward each other, indicating reduced polarization. In contrast, the medium-voltage redox (Nb^4+^/Nb^3+^) peaks gradually shift to lower voltage and diminish in intensity, suggesting poor reversibility. This observation is further supported by the differential capacity curves, which shows that this redox process nearly vanishes after 25 cycles (Figure S6a).

**Figure 3:**
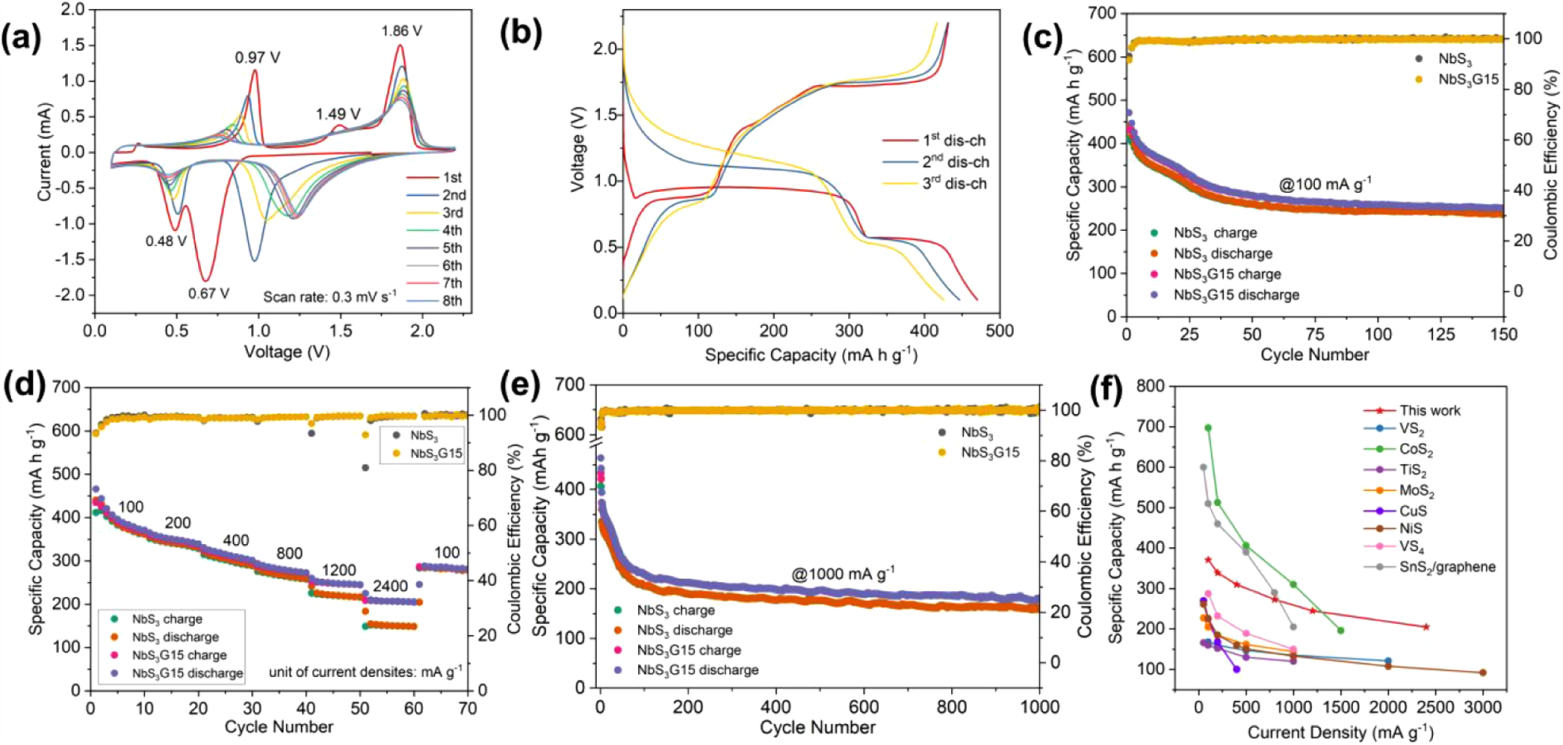
Evaluations of electrochemical performance: (a) The initial eight CV curves of NbS_3_G15 electrodes in MLIBs at a scan rate of 0.3 mV s^–1^ between 0.1 and 2.2 V. (b) The first three (dis)­charge curves of the NbS_3_G15 electrodes in MLIBs at a current density of 100 mA g^–1^. (c) Galvanostatic cycling performance of NbS_3_G15 and NbS_3_ electrodes in MLIBs at 100 mA g^–1^. Capacities of NbS_3_G15 were determined based on the mass of NbS_3_ in the composite. (d) Rate performance of NbS_3_G15 and NbS_3_ electrodes at various current densities. (e) Long-term cycling performance at an elevated current density of 1000 mA g^–1^. (f) Comparison of rate performance between NbS_3_G15 and other selected TMC electrode materials for MLIBs in the literature.

The first three (dis)­charge curves of NbS_3_G15 electrodes in APC and LiAPC electrolytes (Figures S5b and 3b) highlight the stark contrast in electrochemical performance. In APC, the electrode delivers negligible capacity (<25 mA h g^–1^) with no discernible features. In contrast, the first discharge curve in LiAPC exhibits two plateaus at *ca*. 0.95 and 0.56 V, delivering a high capacity of *ca*. 470 mA h g^–1^ (Figure 3b). The subsequent charge curve shows a capacity of *ca*. 431 mA h g^–1^ with three plateaus at *ca.* 0.87 V, 1.42 and 1.73 V, along with a sloped region between 1.45 and 1.70 V. The following cycles reveal a gradual enhancement of the high-voltage plateaus/sloped region, accompanied by the diminution of medium-voltage plateaus, indicating the fading of medium-voltage redox during extended cycling (Figure S6b). The initial three (dis)­charge curves of the NbS_3_ electrode in MLIBs and the NbS_3_G15 electrode in LIBs are provided in Figure S7. This comparison demonstrates that the discharge and charge profiles of NbS_3_G15 or NbS_3_ in both systems are nearly identical, aside from a voltage shift of ∼ 0.8 V due to the lower reduction potential of lithium metal (- 3.04 V vs −2.37 V for magnesium metal; both values vs SHE). These results suggest that Li^+^ ions are the dominant charge carriers responsible for the reversible storage capacity in MLIBs.

The role of graphene in the cycling performance of NbS_3_ in MLIBs was investigated at a current density of 100 mA g^–1^, as shown in Figure 3c. Both NbS_3_G15 and NbS_3_ electrodes (with specific loadings listed in Table S1) exhibit similar trends, showing capacity fading in the initial several tens of cycles before stabilizing. The NbS_3_G15 electrode retains a capacity of *ca.* 251 mA h g^–1^ with 58.1% retention after 150 cycles, slightly outperforming NbS_3_, which achieves a capacity of *ca.* 237 mA h g^–1^ with 57.3% retention. These results suggest that graphene improves electrical conductivity and charge transfer, increasing active sites for ion transport and storage. However, it does not appear to mitigate initial capacity fading or markedly enhance long-term capacity retention. The estimated standard deviations, (251 ± 7) mA h g^–1^ for NbS_3_G15 and (237 ± 6) mA h g^–1^ for NbS_3_, further indicate that the improvement in capacity at low current density is modest and may fall within the range of experimental uncertainty. This suggests that intrinsic structural limitations of NbS_3_, such as irreversible structural/redox changes and material exfoliation/detachment (discussed latter), are the primary contributors to the observed capacity decay. Additionally, the cycling capacity in MIBs is negligible (*ca*. 5–10 mA h g^–1^; Figure S5c), compared to the values observed in MLIBs.

Variable rate measurements (Figure 3d) reveal superior rate performance for NbS_3_G15 compared to NbS_3_, particularly at high current densities. The NbS_3_G15 electrode delivers capacities of *ca.* 371 mA h g^–1^, 339 mA h g^–1^, 310 mA h g^–1^, 273 mA h g^–1^, 245 mA h g^–1^, and 205 mA h g^–1^ at current densities of 100 mA g^–1^, 200 mA g^–1^, 400 mA g^–1^, 800 mA g^–1^, 1200 mA g^–1^, and 2400 mA g^–1^, respectively. In contrast, the NbS_3_ electrode exhibits lower capacities of *ca.* 217 mA h g^–1^ and 149 mA h g^–1^ at 1200 mA g^–1^ and 2400 mA g^–1^, respectively. The enhanced rate performance of NbS_3_G15 is likely attributed to the presence of graphene, which improves overall electrical conductivity and charge transfer, considering the relatively high resistivity of pure NbS_3_ (∼ 100 Ω cm).^44^ Representative (dis)­charge curves under varying current densities for both electrodes are provided in Figure S8 for the comparison of the profiles and capacities. Long-term cycling performance was also evaluated at 1000 mA g^–1^ (Figure 3e). The NbS_3_G15 electrode retains *ca.* 181 mA h g^–1^ with 41.6% capacity retention after 1000 cycles, while NbS_3_ shows a slightly lower capacity of 160 mA h g^–1^ with 39.8% retention. These results further suggest that the effect of graphene on long-term cycling performance is rather modest, and that improving the cyclability of NbS_3_ will require bulk-level modifications, such as doping, to enhance structural stability and redox reversibility. A comparison of variable rate performance with other TMC-based cathodes in MLIBs (Figure 3f) demonstrates that NbS_3_G15 outperforms existing chalcogenide cathodes at high current densities.^11,12,24,45–49^ For example, NbS_3_G15 delivers higher capacities than CoS_2_ and SnS_2_ at 1000 mA g^–1^ and 1500 mA g^–1^. In contrast to conversion-type cathodes, which often suffer from structural reconstruction and significant volume changes, the intercalation mechanism of NbS_3_G15 enables improved structural stability and sustained performance.^50,51^

The morphological and compositional evolution of NbS_3_G15 electrodes at different discharge–charge states was investigated using SEM-EDS. Initially, the uncycled NbS_3_G15 electrode appears as agglomerations of micron- and submicron-sized particles (Figure 4a). Upon the first discharge to 0.8 V, the morphology changes dramatically (Figure 4b): bulk particles transform into bundles of nano needles with high aspect ratios (widths of 100–300 nm). Further discharge to 0.1 V results in exfoliation and separation of the nano needles from the bulk particles (Figure 4c). After the first charge, the electrode displays shorter needles and small detached crystals dispersed around the bulk material (Figure 4d), as highlighted by white rectangles and arrows. Continued cycling leads to further needle formation and progressive thinning of the bulk particles (Figures 4e,f). This morphological evolution indicates volume expansion of the NbS_3_ structure induced by the incorporation of Li^+^ and/or magnesium ions, consistent with similar phenomena observed in transition metal trichalcogenides such as TiS_3_, HfS_3_, and ZrS_3_ upon chemical lithiation with *n*-butyllithium.^52,53^ One possible hypothesis for the observed microstructural evolution is as follows: Similar to other trichalcogenides, the quasi-1D chain structure of NbS_3_, comprising [NbS_6_]_*n*_ chains extending along the crystallographic *b*-axis and assembled via weak covalent bond along the *a-*axis, facilitates cleavage along the chain direction. For instance, TiS_3_ has been reported to exhibit low theoretical cleavage energies of 0.320, 0.714, 0.716, and 0.815 J m^–2^ along (001), (101), (101̅), and (100) planes, respectively, enabling separation of pseudolayers and [TiS_6_]_*n*_ chains. In contrast, the (010) plane exhibits a much higher cleavage energy of 2.706 J m^–2^, making exfoliation along that direction less favorable. Similarly, chemically lithiated ZrS_3_ has been shown to undergo significant expansion along the *a*-axis (i.e., the width direction) due to intercalation-induced stress, while its length along the *b*-axis remains nearly unchanged.^53^ These insights suggest that Li^+^ intercalation into NbS_3_ likely induces expansion of the [NbS_6_]_*n*_ interchain, promoting exfoliation of bulk particles into bundles of needle-like crystallites. This exfoliation-driven nanosizing could shorten ionic diffusion pathways, enhance Li^+^ and/or magnesium ion transport, and promote substantial (near) surface pseudocapacitance storage. However, the exfoliation process may lead to partial particle detachment from the conductive matrix, contributing to capacity loss in the initial cycles. SEM images of the NbS_3_G15 electrodes after the 25th discharge/charge cycle (Figure S9) show that the bulk particles have undergone substantial exfoliation, leaving slender crystals dispersed throughout the electrode. Those located on the electrode surface appear to accumulate as electrically disconnected “dead” particles.

**Figure 4:**
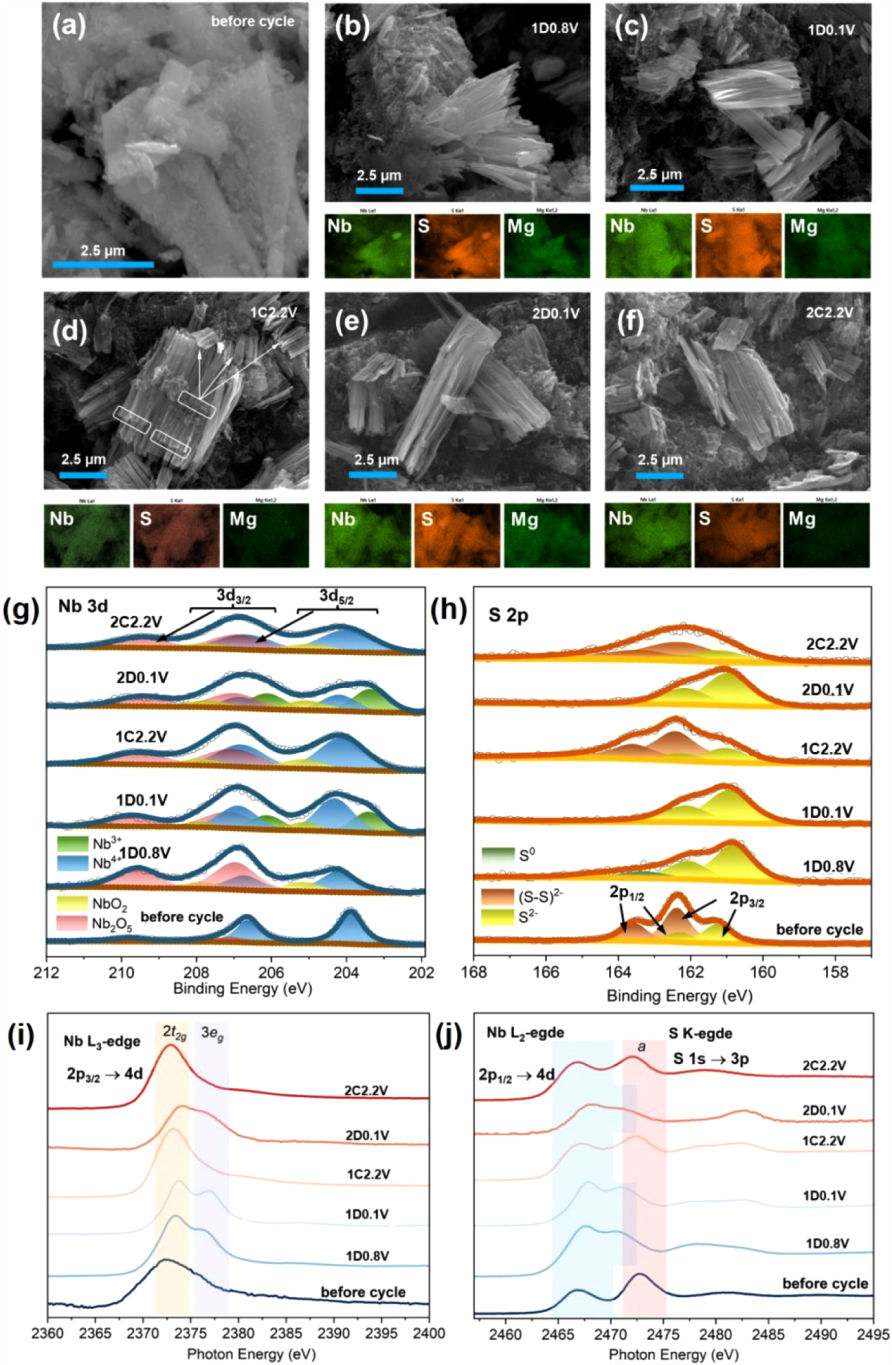
SEM-EDS, XPS, and XAS studies of NbS_3_G15 electrodes at various states: SEM image (a) before cycling, and SEM-EDS elemental map images after (b) the 1st discharge 0.8 V (1D0.8 V), (c) 1D0.1 V, (d) 1C2.2 V, (e) 2D0.1 V, and (f) 2C2.2 V states in MLIBs. High-resolution *ex situ* XPS spectra of (g) Nb 3d and (h) S 2p transitions, and *ex situ* XAS spectra of (i) Nb L_3_-edge and (j) Nb L_2_-edge and S K-edge of NbS_3_G15 electrodes at different (dis)­charge states.

The EDS elemental maps for Nb, S, and Mg at various discharge–charge states (Figures 4b_‑_f), along with the corresponding EDS spectra (Figure S10), reveal uniform distributions of Nb, S, and Mg across the exfoliated crystals. Magnesium concentration decreases upon charging and is replenished during discharging. Considering the nearly identical discharge–charge profiles of the NbS_3_G15 electrode in MLIB and LIB systems, the Mg-related capacity is likely dominated by (near) surface insertion/adsorption, i.e., pseudo capacitive behavior, promoted by the exfoliation-induced nanosizing of NbS_3_ particles during cycling. Based on the detailed EDS composition data (Table S1), the Mg:Nb atomic ratio after the first discharge is ∼ 0.32 (corresponding to a composition of Li_2.36_Mg_0.32_NbS_3_, equivalent to a Mg-related capacity of ∼ 91 mA h g^–1^), which decreases to ∼ 0.14 after charging. To further decouple the Mg^2+^ contribution, the NbS_3_G15 electrode was first cycled once to the charged state in MLIB, then removed, washed, reassembled, and cycled in a Mg-only (MIB) system. As shown in Figure S11, the activated NbS_3_G15 electrode in MIB exhibits discharge–charge profiles that partially overlap with those in MLIB and delivers an average capacity of 94 mA h g^–1^, indicating dominant Li^+^ intercalation (88.1%) with a minor Mg^2+^ contribution (11.9%). In the second cycle, a higher Mg:Nb ratio of ∼ 0.52 is observed at discharged state, dropping to ∼ 0.16 upon charging, suggesting partial retention of magnesium ions in the host lattice after each cycle. Notably, the Cl/Mg ratios in cycled electrodes are much lower than those expected for intercalating magnesium-based species such as MgCl^+^ and Mg_2_Cl_3_^+^. Instead, the Cl/Al ratios match well with AlCl_4–*n*_Ph_*n*_^–^ (*n* = 0–4) species originating from the APC electrolyte.^54^ This indicates that Mg^2+^ is the primary magnesium intercalant, while Cl and Al result from surface electrolyte adsorption. At the 25th discharged and charged states (Figure S12 and Table S2), reversible magnesium ion cointercalation persists. However, the concentrations of Mg and Cl become comparably large and exhibit similar trends with cycling, indicating that cointercalated magnesium species have transitioned into Mg_*x*_Cl_*y*_^+^ over extended cycling. This transformation may result from cycling-induced effects, such as nano sizing and structural rearrangements in the diffusion pathways, which facilitate the insertion of larger cationic species. The Mg:Nb atomic ratio at the 25th discharged state reaches *ca.* 0.7, corresponding to a Mg_*x*_Cl_*y*_^+^ capacity of *ca.* 99 mA h g^–1^, approximately half of the capacity attributed to Li^+^ ions.

*Ex situ* XPS and XAS were employed to investigate the changes in the oxidation states of Nb and S in the NbS_3_G15 electrodes during cycling. Figure 4g,h show the high-resolution Nb 3d and S 2p spectra, respectively, for the as-prepared NbS_3_G15 and samples at various (dis)­charge states (see Table S3). Upon the first discharge to 0.8 V (1D0.8 V), no significant shift in Nb 3d binding energies toward lower values is observed. However, paired peaks at *ca.* 207.0/209.6 eV and *ca.* 205.1/207.9 eV emerge, corresponding to Nb^5+^ and Nb^4+^ from surface oxides Nb_2_O_5_ and NbO_2_, respectively, likely formed during sample preparation and transfer, an occurrence common in intercalated chalcogenides.^55^ Concurrently, the S_2_^2–^ doublet peaks at *ca*. 162.4 and 163.6 eV disappear, while the S^2–^ peaks at *ca.* 160.8 and 162.1 eV intensify, indicating the reduction of S­(−I) to S­(−II). This suggests that during initial lithiation, S_2_^2–^ species are primarily reduced to S^2–^, while Nb remains largely in the +4 oxidation state. This is consistent with a two-electron transfer per NbS_3_ unit, assuming disulfide anions are reduced and the third sulfur is already S^2–^. Further discharge to 0.1 V results in the growth of peaks at *ca.* 203.4 and 206.1 eV in the Nb spectrum, corresponding to Nb^3+^ in sulfides,^56,57^ with minimal changes in the S 2p region, confirming the reduction of Nb^4+^ to Nb^3+^. Upon charging to 2.2 V, Nb^4+^ (204.2 and 206.9 eV) and S­(−I) peaks are nearly restored, confirming the reversibility of both Nb and S redox processes. Similar trends are observed in the second cycle. It is worth noting that Li–Mg–Nb-S phases, like other alkali and alkaline-earth metal-intercalated chalcogenides, are highly sensitive to air and moisture. Even minor exposure can lead to oxidation or hydrolysis, resulting in the formation of NbO_2_, Nb_2_O_5_, elemental sulfur, or metal hydroxides, which may complicate the XPS data interpretation.

*Ex situ* XAS spectra at the Nb L-edge (Figure 4i) and S K-edge (Figure 4j) further corroborate the oxidation state changes discussed above. The Nb L_3_ absorption edge (2367–2385 eV) arises from the dipole-allowed transition from Nb 2p_3/2_ to 4d orbitals, while the L_2_-edge (2462 −2473 eV) corresponds to the transition from 2p_1/2_ to 4d orbitals.^58^ In the S K-edge spectra, the singlet peak *a* (highlighted by the light red band) represents the transition from S 1s to 3p orbitals.^59^ The spectral features of both the Nb L-edge and S K-edge evolve with (dis)­charge state. Upon discharge to the 1D0.8 V state, the Nb L_3_-edge spectrum shows the emergence of an *e*_g_ peak at *ca.* 2376.8 eV, while the intensity of peak *a* in the S K-edge spectrum decreases markedly. These changes indicate the reduction of disulfide (S_2_^2–^) to sulfide (S^2–^) anions.^60,61^ The slight upshift in the *t*_*2g*_/*e*_g_ peaks may reflect significant local structural changes around niobium, likely due to the sulfur anion reduction. This may induce a coordination shift from trigonal prismatic to octahedral symmetry, thereby increasing the crystal field splitting energy. Full discharge results in a reduced *t*_*2g*_/*e*_g_ peak intensity ratio in the Nb L_3_-edge spectrum, while the S K-edge spectrum remains largely unchanged. This suggests the injection of electrons to Nb *t*_2g_ orbitals, which lowers the population of unoccupied states available for electronic transitions, indicating the reduction of Nb^4+^ to Nb^3+^. Upon charging, the Nb L_3_-edge spectrum shows a return of the *t*_*2g*_ peak to an energy position comparable to that in the pristine state, and peak *a* in the S K-edge spectrum is nearly fully recovered, confirming the reversibility of both the S_2_^2–^/S^2–^ and Nb^4+^/Nb^3+^ redox reactions. Similar spectral changes are observed in the second cycle, reinforcing the participation of both cationic and anionic redox in the cycling mechanism. Additionally, some changes in the postedge region (2477–2483 eV) are noted. These features likely arise from S 1s transitions to higher-energy hybridized orbitals (e.g., S 3p-Nb 5s), along with possible contributions from Nb 2p_1/2_ to ligand field states (e.g., 3*a*_1g_) in both pristine sulfides and randomly oxidized sulfide environments.^58,62^ However, due to overlapping electronic transitions, surface oxidation effects, and the limited availability of references in the literature, definitive peak assignment and analysis remains challenging.

The structural evolution of the NbS_3_G15 electrode in MLIBs was investigated using *in operando* PXRD. Figure 5a presents the PXRD patterns collected during the first two (dis)­charge cycles at a current density of 100 mA g^–1^. During the first discharge to 0.65 V (highlighted by the solid red circle), corresponding to a two-electron transfer, the co-intercalation of Li^+^ and Mg^2+^ ions leads to a progressive disappearance of diffraction peaks above 30° (all 2θ here and below), along with a reduction in intensity of the prominent peaks at *ca*. 9.7° (001) and 19.5° (002), as indicated by dark blue arrows. Subsequently, new peaks emerge at *ca*. 13.8°, 35.0°, 48.5°, and 50.2° (highlighted by yellow arrows), while the positions of the (001) and (002) reflections remain unchanged. These changes suggest the formation of a poorly crystalline intercalated phase (phase 1), which likely retains the 1D [NbS_6_]_*n*_ chain structure, consistent with the needle-like morphologies observed by SEM in the discharged samples. As shown in Figure S13, phase 1 at the 1D0.65 V state, with a composition approximated as Li_*a*_Mg_(10.5*a*)_NbS_3_ (*a* < 2; fully intercalated/adsorbed composition: Li_1.44_Mg_0.28_NbS_3_), exhibits significant PXRD pattern mismatches with the monoclinic and cubic Li_2_NbS_3_ phases. This indicates that NbS_3_ is unlikely to transform into these phases. Otherwise, the formation of these structures (Figure S14), distinct from the chain-like NbS_3_ compound, would likely disrupt the strong intrachain S–Nb–S bonding and potentially break the 1D needle-like morphology of the electrode. Furthermore, since monoclinic Li_2_TiS_3_ has been reported to be electrochemically inactive in LIBs,^63^ a structurally similar monoclinic Li_2_NbS_3_ phase would be expected to exhibit comparable inactivity, which contrasts with the reversible electrochemical behavior observed for intercalated NbS_3_ in this study. Further discharge to 0.1 V (1D0.1 V) results in additional structural changes: the peak at *ca.* 35.0° disappears, two new peaks appear at *ca.* 32.2° and 38.3°, and the peak at *ca.* 13.8° shifts to a lower angle. The (001) peak at *ca*. 9.7° intensifies slightly but remains at the same 2θ position. These changes indicate the formation of a second phase (phase 2), with an approximate composition of Li_*b*_Mg_(1.50.5*b*)_NbS_3_ (*b* < 3; fully inserted/adsorbed composition: Li_2.36_Mg_0.32_NbS_3_). The PXRD pattern of phase 2 (peaks highlighted by white arrows) closely resembles that of a previously reported Li_3_TiS_3_-like structure (unassigned), formed by chemical lithiation of TiS_3_ using *n*-butyllithium.^42^ Similar to Li_3_TiS_3_, the 1D chain framework in phase 2 is likely preserved, consistent with the needlelike morphology observed in the electrode particles.

**Figure 5:**
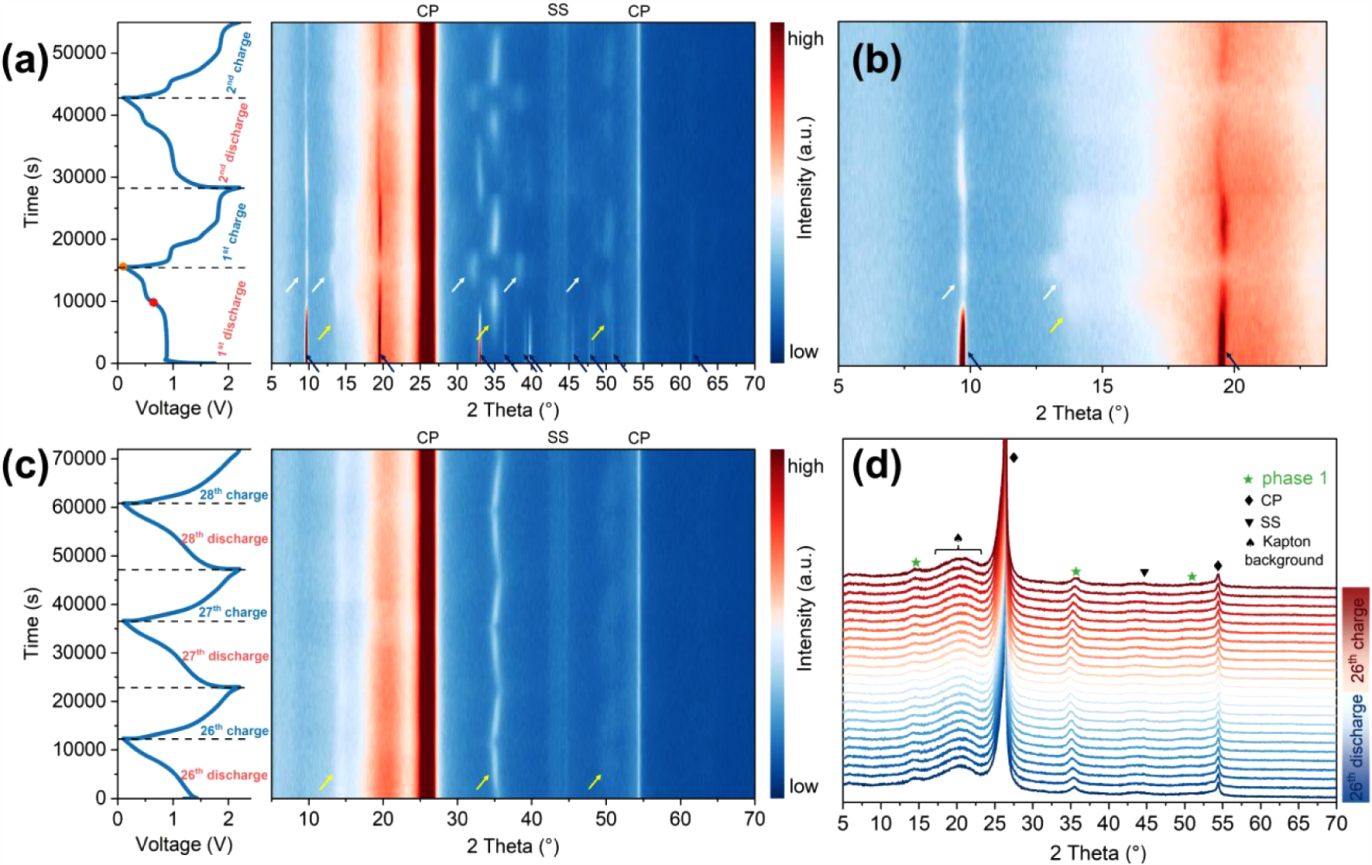
(a) The first two cycles of time-resolved discharge–charge curves (left; solid red and orange circles: 1D0.65 V and 1D0.1 V states, respectively) and the corresponding contour plot of *in operando* PXRD patterns (right) for an NbS_3_G15 electrode in an MLIB cell cycling at a current density of 100 mA g^–1^. The diffraction peaks for NbS_3_, phases 1 and 2 are highlighted by dark blue, yellow, and white arrows, respectively. (b) Zoomed PXRD region taken from (a). (c) *In operando* PXRD patterns (right) for an NbS_3_G15 electrode in a MLIB cell from the 26th to 28th cycles at a current density of 100 mA g^–1^. (d) Stacking plot of *in operando* PXRD patterns taken during extended cycles. Reflections for the carbon paper current collector (CP) and the stainless-steel coin cell case (SS) are indicated in the plots.

The lattice expansion observed in phases 1 and 2 appears to occur primarily within the interchain region, as indicated by the unchanged positions of the (001) and (002) reflections associated with the pseudolayers (Figure 5b). This interchain expansion is attributable to the structural adjustments in the [NbS_6_]_*n*_ chains upon incorporating Li^+^/Mg^2+^ ions, which may be driven by several factors. First, Li^+^/Mg^2+^ intercalation likely disrupts the weak interchain interactions, enabling ions to occupy octahedral or tetrahedral interstitial sites.^64^ This leads to expansion along the *a*-axis, where the chains are aligned, while the vdWs gaps between pseudolayers remain largely unaffected. Second, the breakage of S–S bonds during discharge may increase electrostatic repulsion between the resulting S^2–^ anions, further contributing to interchain expansion along the *a*-axis.^64^ These deductions are consistent with observations of interchain expansion in chemically lithiated TiS_3_ with a composition of Li_3_TiS_3_.^52^

During the first charge, phase 2 reversibly transitions back to phase 1 as Li^+^/Mg^2+^ ions equivalent to one electron per formula unit are deintercalated. The diffraction peaks associated with phase 1 subsequently shift slightly to higher angles, followed by the recovery of a poorly crystalline NbS_3_ phase. In the second cycle, the NbS_3_G15 electrode undergoes similar structural changes; however, the peak intensities of phase 2 and recovered NbS_3_ decrease, indicating increasing structural irreversibility. These observations suggest that phase 1 emerges as the dominant reversible structure during prolonged cycling, which will be analyzed in the following discussion.

Additionally, *in operando* PXRD patterns of the NbS_3_G15 electrode in LIB were collected to analyze the effect of Li^+^/Mg^2+^ insertion/adsorption. As shown in Figure S15, the changes in the peak positions and intensities are nearly identical to those observed in MLIB, suggesting that Mg^2+^ coinsertion/adsorption has negligible influence on the structural evolution induced by Li^+^ insertion.

Further *in* *operando* PXRD analysis of the NbS_3_G15 electrode at the 25th charged state provides additional insights into its long-term structural evolution (Figure 5c). The PXRD patterns reveal persistent diffraction peaks corresponding to phase 1 (*ca*. 13.8°, 35.0°, and 50.2°, highlighted by yellow arrows), while reflections associated with phase 2 and the pristine NbS_3_ phase are absent. Notably, the (001) and (002) reflections at *ca*. 9.7° and 19.5°, respectively, also disappear, likely due to the loss of long-range order within the pseudolayers and/or reduction in crystallite size from exfoliation during repeated (de)­intercalation of Li^+^/Mg_*x*_Cl_*y*_^+^ ions (as confirmed by EDS analysis, which indicates a shift from Mg^2+^ to Mg_*x*_Cl_*y*_^+^ species over cycling). The diffraction peak at *ca.* 35.4° exhibits a gradual shift to lower 2θ values during discharge (expansion) and returns to higher angles upon charging (contraction), consistent with reversible lattice adjustments associated with Li^+^/Mg_*x*_Cl_*y*_^+^ intercalation/deintercalation. To emphasize the evolving peak profiles, a stacked plot of the PXRD patterns from the 26th cycle is shown in Figure 5d. The *in operando* PXRD data from both initial and extended cycles reveal key structural factors underlying the capacity fading observed in Figure 3c. The disappearance of phase 2 and the absence of reflections for NbS_3_ suggest the loss or suppression of their associated redox reactions. Notably, the theoretical capacity contribution of phase 2 (*ca.* 142 mA h g^–1^) closely corresponds to the observed capacity loss (*ca.* 146 mA h g^–1^) by the 26th cycle, reinforcing its critical role in the initial high capacity and its subsequent degradation as a key factor in performance decline.

To help elucidate the structural evolution of NbS_3_ in the NbS_3_G15 electrode during both initial and extended cycles, a hypothetical schematic is proposed and presented in Figure 6. The first discharge to 0.65 V and subsequently to 0.1 V leads to the formation of phases 1 and 2, respectively. In both phases, the interchain spacing is expanded—evidenced by the emergence of the diffraction peak at *ca.* 13.8° and its further shift to lower angle upon deeper discharge—due to the accommodation of Li^+^ and Mg^2+^ cations and associated local structural adjustments. These include changes in Nb–S bond lengths and electrostatic interactions between neighboring S^2–^ anions formed from the reduction of S_2_^2–^. Upon charging, the extraction of Li^+^/Mg^2+^ ions induces a reversal of these changes. However, with repeated cycling, the electrode material undergoes intensive exfoliation, resulting in reduced particle size, while the pseudolayers become increasingly disordered. It is therefore hypothesized that, during extended cycling, structural expansion and contraction proceed primarily within the alternative slab (or pseudolayer) highlighted by the blue dashed rectangle.

**Figure 6:**
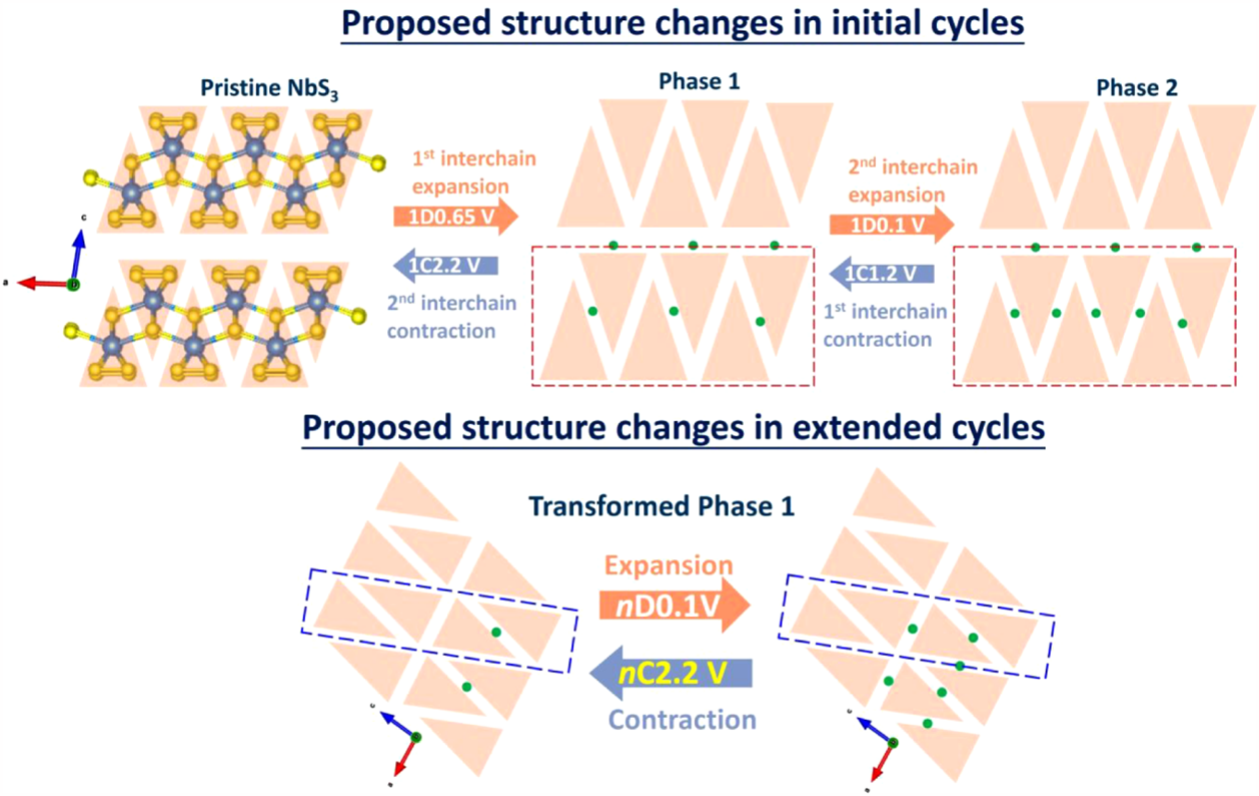
A simplified schematic illustrating the hypothesized structural evolution of NbS_3_ in the NbS_3_G15 electrode during initial and extended cycles. The [NbS_6_]_*n*_ chains, extending along the *b*-axis and aligned along the *a-*axis, are represented as light orange triangles. Green solid circles indicate intercalated Li^+^/Mg^2+^ (or Mg_*x*_Cl_*y*_^+^) ions. The red dashed rectangle denotes the pseudolayer structure in the initial cycles, while the blue dashed rectangle highlights the proposed “pseudo-layer” or “slab” of the transformed phase 1 structure during the extended cycling.

The electrochemical properties of NbS_3_G15 were further investigated using CV, the galvanostatic intermittent titration technique (GITT), and electrochemical impedance spectroscopy (EIS). To assess the charge storage behavior semi-quantitatively, CV curves were collected at various scan rates (0.1 mV s^–1^, 0.3 mV s^–1^, 0.5 mV s^–1^, 0.7 mV s^–1^, and 0.8 mV s^–1^), analyzed, and fit linearly according to Equations S1 and S2. The resulting plots are shown in Figure 7a,b. The calculated *b*-values for the two reductive peaks and one oxidative peak were 0.795 ± 0.026, 0.706 ± 0.030, and 0.818 ± 0.024, respectively. These intermediate values (between 0.5 and 1.0) suggest a mixed charge storage mechanism involving both capacitive and diffusion-controlled processes. Further quantification of the capacitive and diffusion-controlled contributions was conducted using the current-scan rate data from the CV curves in Figure 7a. By the linear fitting of *i*/*v* ^1/2^ vs 1/*v* ^1/2^ (Equation S3), the constants *k*_1_ and *k*_2_ were determined. The results reveal substantial capacitative contributions of 91%, 92%, 93%, 95%, and 96% at scan rates of 0.1 mV s^–1^, 0.3 mV s^–1^, 0.5 mV s^–1^, 0.7 mV s^–1^, and 0.8 mV s^–1^, respectively (Figure 7c). This demonstrates that the charge storage mechanism in NbS_3_G15 is predominantly governed by fast, (near-)­surface pseudocapacitive processes. Such behavior is likely attributed to the significant morphological evolution of the electrode during cycling, wherein bulk NbS_3_ particles are transformed into nanoscale needle-like crystals (Figure 4a–f). This transformation enhances the surface area and increases the number of accessible active sites for rapid surface charge storage, while also improving ion diffusion within the nanosized domains. Consequently, the electrode supports intercalation pseudocapacitance with minimal structural disruptions, as evidenced by the relatively stable PXRD patterns and morphologies after extended cycling (see Figures 5c,d and S9).^65–67^

**Figure 7:**
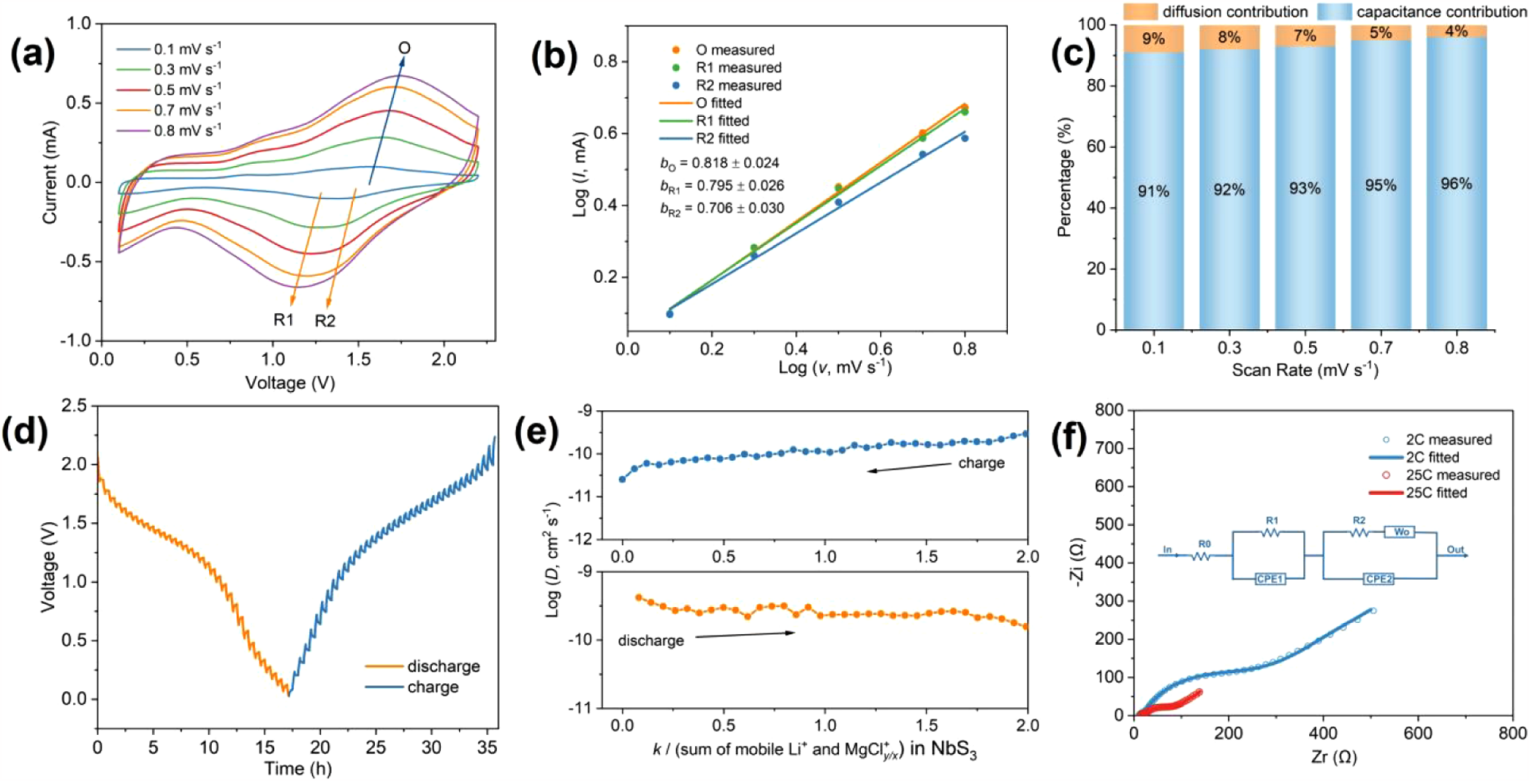
(a) CV curves of the NbS_3_G15 electrode recorded at scan rates of 0.1 mV s^–1^ (blue), 0.3 mV s^–1^ (green), 0.5 mV s^–1^ (red), 0.7 mV s^–1^ (orange), and 0.8 mV s^–1^ (purple). (b) Log–log plots of oxidation (orange) and reduction (green and blue) peak currents vs scan rate (*v*), with solid lines indicating linear fits. (c) Histogram showing the capacitative and diffusive contributions to the total current at different scan rates. (d) GITT curves of the NbS_3_G15 electrode at a current density of 50 mA g^–1^. (e) Plots of calculated Li^+^/Mg_*x*_Cl_*y*_^+^ diffusion coefficients during discharge and charge as a function of the mixed cation intercalation level (*k*). (f) Measured (circles) and fitted (solid lines) EIS spectra of NbS_3_G15 electrodes at the 2nd charge and 25th charge states in Mg|LiAPC|NbS_3_G15 cells. The equivalent circuit model is shown in the inset.

The GITT measurements, described in the Supporting Information, provide insights into the mixed-ion diffusion kinetics of Li^+^ and Mg_*x*_Cl_*y*_^+^ in the NbS_3_G15 electrode. From the GITT curves (Figure 7d), effective diffusion coefficients (*D*) were calculated using Equation S4 and plotted as a function of the total number of mixed ions *k* (where *k* = *n* + *m* in the composition Li_*n*_(MgCl_*y/x*_)_*m*_NbS_3_) at various discharge–charge states (Figure 7e), reflecting the overall mass transport kinetics of the mixed ion system. During discharge, the *D* values range from 1.16 × 10^–10^ cm^2^ s^–1^ to 4.19 × 10^–10^ cm^2^ s^–1^, while during charge, they range from 2.53 × 10^–11^ cm^2^ s^–1^ to 4.16 × 10^–10^ cm^2^ s^–1^. The average discharge diffusion coefficient, 2.5 × 10^–10^ cm^2^ s^–1^, is comparable to or even exceeds those reported for other cointercalation chalcogenide electrode materials in MLIBs, such as VS_4_ (8.3 × 10^–10^ – 6.3 × 10^–9^ cm^2^ s^–1^),^25^ TiS_2_ (1 × 10^–10^ – 10^–9^ cm s^–1^),^46^ and MoS_2_ (3.1 × 10^–13^ – 3.1 × 10^–11^ cm s^–1^).^10^ These results suggest that the NbS_3_G15 electrode enables relatively efficient mixed ion transport while maintaining high capacities.

EIS was conducted to evaluate charge transfer resistance, interfacial properties, and ion diffusion processes in the NbS_3_G15 electrode. Nyquist plots of the uncycled electrode, and those after the second and 25th charge cycles were fitted using a modified Randles circuit model using Aftermath software^68^ (Figures S17a and 7f). The equivalent circuit consists of three segments: (1) R_0_, representing internal resistance from the electrolyte and current collector, among others; (2) the high-frequency region, modeled as a parallel combination of resistance and constant phase element (R_1_ + CPE_1_), corresponding to the solid-electrolyte-interphase (SEI); and (3) the low-frequency region, modeled as a parallel pair of (R_2_ + W_0_) & CPE_2_, representing mixed charge transfer and diffusion-controlled processes. The extracted parameters (Table S4) show a significant reduction in SEI resistance (R_1_) from 842 Ω (uncycled), to 9 Ω (the second cycle), which then stabilizes at 11 Ω after 25th cycle, indicating initial surface modifications (e.g., removal of the oxidation/passivation layers) and subsequent SEI stabilization. Similarly, R_2_ and W_0_ decrease markedly from 4737 Ω and 609 Ω s^–0.5^ (uncycled) to 238 Ω and 280 Ω s^–0.5^ (the second cycle), and further to *ca.* 56 Ω and 59 Ω s^–0.5^ (the 25th cycle). These reductions highlight enhanced charge transfer and improved mixed ion diffusion with cycling, consistent with the “activation process” commonly reported in the literature.^22,69,70^ Additionally, the NbS_3_G15 electrode exhibits obviously lower R_1_ and R_2_ values before and after cycling compared to the graphene-free NbS_3_ electrode (Figure S17b,c), indicating enhanced electrical conductivity facilitated by graphene compositing, and thus improved ion migration at the interphase and charge transfer on the NbS_3_ surface.

## 4 Conclusions

In summary, this study explores the application of PVT-synthesized NbS_3_, incorporated with graphene, as a novel cathode material for MLIBs. The NbS_3_-graphene composite exhibits promising electrochemical performance, delivering high capacities at both low and high rates (*ca.* 251 mA h g^–1^ after 150 cycles at 100 mA g^–1^ and *ca.* 181 mA h g^–1^ after 1000 cycles at 100 mA g^–1^), as well as excellent rate capability (205 mA h g^–1^ at 2400 mA g^–1^). Despite the above impressive performance, the capacity retentions at low and high current densities remain similar between NbS_3_G15 and NbS_3_ electrodes (58.1% vs 57.3% at 100 mA g^–1^; 41.6% vs 39.8% at 1000 mA g^–1^), suggesting that the incorporation of graphene provides only modest enhancement in cycling stability. The observed capacity fade is therefore primarily attributed to intrinsic structural limitations of bulk NbS_3_. Mechanistic analysis, the core of this work, reveals that the fade behavior is governed by increasingly irreversible structural changes and cationic redox processes in the medium voltage range. *in operando* PXRD identifies two-step phase transitions during initial cycling, involving Li^+^/Mg^2+^ co-intercalated phases 1 and 2, which are likely associated with sequential S_2_^2–^/S^2–^ and Nb^4+^/Nb^3+^ redox reactions, as corroborated by *ex situ* XPS and XAS. With extended cycling, however, only phase 1—corresponding to reversible S_2_^2–^/S^2–^ redox—persists as an active host, while phase 2 and the original NbS_3_ structure gradually fade. The decay of phase 2 and the associated cationic redox coincides with the major capacity drop observed within the first tens of cycles. Additionally, exfoliation-induced detachment of active material, confirmed by *ex situ* SEM, further contributes to capacity loss and instability in initial cycling. These findings highlight the central role of reversible disulfide redox chemistry in enabling high-capacity MLIB cathodes and point to the necessity of bulk structural engineering in future work, such as doping, to enhance phase stability and redox reversibility, thereby achieving stable cycling and higher capacity. This work not only provides a comprehensive understanding of the fundamental electrochemical behavior and energy storage mechanisms of NbS_3_-based materials but also underscores the broader potential of redox-active disulfide-containing chalcogenides as viable candidates for next-generation, high-capacity Mg-based batteries.

## Supplementary Material



## Notes

PXRD patterns, crystal structures, SEM images, EDS spectra, TG-DSC curves, CV curves, (dis)­charge curves, and EIS spectrum (PDF)

## References

[1] Zeng X., Li M., Abd El-Hady D., Alshitari W., Al-Bogami A. S., Lu J., Amine K. (2019). Commercialization of Lithium Battery Technologies for
Electric Vehicles. Adv. Energy Mater..

[2] Mu T., Wang Z., Yao N., Zhang M., Bai M., Wang Z., Wang X., Cai X., Ma Y. (2023). Technological
penetration and carbon-neutral evaluation of rechargeable battery
systems for large-scale energy storage. J. Energy
Storage.

[3] Liu F., Wang T., Liu X., Fan L.-Z. (2021). Challenges and Recent
Progress on Key Materials for Rechargeable Magnesium Batteries. Adv. Energy Mater..

[4] Canepa P., Sai Gautam G., Hannah D. C., Malik R., Liu M., Gallagher K. G., Persson K. A., Ceder G. (2017). Odyssey of Multivalent
Cathode Materials: Open Questions and Future Challenges. Chem. Rev..

[5] Maroni F., Dongmo S., Gauckler C., Marinaro M., Wohlfahrt-Mehrens M. (2021). Through the
Maze of Multivalent-Ion Batteries: A Critical Review on the Status
of the Research on Cathode Materials for Mg^2+^ and Ca^2+^ Ions Insertion. Batteries Supercaps.

[6] Chang H. J., Cheng Y., Choi D., Dong H., Li G., Liu J., Sprenkle V. L., Yao Y. (2016). Rechargeable Mg–Li hybrid
batteries: status and challenges. J. Mater.
Res..

[7] Mao M., Gao T., Hou S., Wang C. (2018). A critical review of cathodes for
rechargeable Mg batteries. Chem. Soc. Rev..

[8] Liang Y., Dong H., Aurbach D., Yao Y. (2020). Current status and
future directions of multivalent metal-ion batteries. Nat. Energy.

[9] Yoo H. D., Liang Y., Li Y., Yao Y. (2015). High Areal
Capacity
Hybrid Magnesium–Lithium-Ion Battery with 99.9% Coulombic Efficiency
for Large-Scale Energy Storage. ACS Appl. Mater.
Interfaces.

[10] Xiong Z., Zhu G., Wu H., Shi G., Xu P., Yi H., Mao Y., Wang B., Yu X. (2022). Hydrochloric Acid-Assisted Synthesis
of Highly Dispersed MoS_2_ Nanoflowers as the Cathode Material
for Mg-Li Batteries. ACS Appl. Energy Mater..

[11] Meng Y., Zhao Y., Wang D., Yang D., Gao Y., Lian R., Chen G., Wei Y. (2018). Fast Li^+^ diffusion in interlayer-expanded vanadium disulfide nanosheets for
Li^+^/Mg^2+^ hybrid-ion batteries. J. Mater. Chem. A.

[12] Zhu G., Xia G., Pan H., Yu X. (2022). Size-Controllable Nickel Sulfide
Nanoparticles Embedded in Carbon Nanofibers as High-Rate Conversion
Cathodes for Hybrid Mg-Based Battery. Adv. Sci..

[13] Wang J., Wang X., Yang J., Dong X., Chen X., Zhang Y., Zeng W., Xu J., Wang J., Huang G. (2022). Core-Shell CuS@MoS_2_ Cathodes for High-Performance
Hybrid Mg-Li Ion Batteries. J. Electrochem.
Soc..

[14] Chen H., Xu H., Li B., Li Z., Zhang K., Zou J., Hu Z., Laine R. M. (2022). Using amorphous CoS_x_ hollow nanocages as
cathodes for high-performance magnesium-lithium dual-ion batteries. Appl. Surf. Sci..

[15] Zhang Y., Xie J., Han Y., Li C. (2015). Dual-Salt Mg-Based Batteries with
Conversion Cathodes. Adv. Funct. Mater..

[16] Zak J. J., Kim S. S., Laskowski F. A. L., See K. A. (2022). An Exploration of
Sulfur Redox in Lithium Battery Cathodes. J.
Am. Chem. Soc..

[17] Xue X., Huang T., Zhang Y., Qi J., Wei F., Sui Y., Jin Z. (2023). Cationic–Anionic
Redox Chemistry in Multivalent
Metal-Ion Batteries: Recent Advances, Reaction Mechanism, Advanced
Characterization Techniques, and Prospects. Adv. Funct. Mater..

[18] Zhang S. S., Tran D. T. (2016). Mechanism and Solution for the Capacity Fading of Li/FeS_2_ Battery. J. Electrochem. Soc..

[19] Kozlova M. N., Mironov Y. V., Grayfer E. D., Smolentsev A. I., Zaikovskii V. I., Nebogatikova N. A., Podlipskaya T. Y., Fedorov V. E. (2015). Synthesis Crystal Structure, and
Colloidal Dispersions
of Vanadium Tetrasulfide (VS_4_). Chem.
Eur. J..

[20] Britto S., Leskes M., Hua X., Hébert C.-A., Shin H. S., Clarke S., Borkiewicz O., Chapman K. W., Seshadri R., Cho J. (2015). Multiple
Redox Modes in the Reversible Lithiation of High-Capacity, Peierls-Distorted
Vanadium Sulfide. J. Am. Chem. Soc..

[21] Pham D. T., Sambandam B., Kim S., Jo J., Kim S., Park S., Mathew V., Sun Y.-K., Kim K., Kim J. (2019). A zero fading sodium ion battery: High compatibility microspherical
patronite in ether-based electrolyte. Energy
Storage Mater..

[22] Wang Y., Liu Z., Wang C., Yi X., Chen R., Ma L., Hu Y., Zhu G., Chen T., Tie Z. (2018). Highly
Branched VS_4_ Nanodendrites with 1D Atomic-Chain Structure
as a Promising Cathode Material for Long-Cycling Magnesium Batteries. Adv. Mater..

[23] Jing P., Lu H., Yang W., Cao Y., Xu B., Cai W., Deng Y. (2020). Polyaniline-coated VS_4_@rGO nanocomposite as high-performance
cathode material for magnesium batteries based on Mg^2+^/Li^+^ dual ion electrolytes. Ionics.

[24] Wang Y., Wang C., Yi X., Hu Y., Wang L., Ma L., Zhu G., Chen T., Jin Z. (2019). Hybrid Mg/Li-ion batteries
enabled by Mg^2+^/Li^+^ co-intercalation in VS_4_ nanodendrites. Energy Storage Mater.

[25] Zhang X., Tu X., Liu Y., Zhu Y., Zhang J., Wang J., Shi R., Li L. (2023). Morphology
Engineering of VS_4_ Microspheres
as High-Performance Cathodes for Hybrid Mg^2+^/Li^+^ Batteries. ACS Appl. Mater. Interfaces.

[26] Halat D. M., Britto S., Griffith K. J., Jónsson E., Grey C. P. (2019). Natural abundance solid-state ^33^S NMR study
of NbS_3_: applications for battery conversion electrodes. Chem. Commun.

[27] Conny J. M., Powell C. J. (2000). Standard test data
for estimating peak parameter errors
in x-ray photoelectron spectroscopy III. Errors with different curve-fitting
approaches. Surf. Interface Anal..

[28] Jain V., Biesinger M. C., Linford M. R. (2018). The Gaussian-Lorentzian Sum, Product,
and Convolution (Voigt) functions in the context of peak fitting X-ray
photoelectron spectroscopy (XPS) narrow scans. Appl. Surf. Sci..

[29] Bloodgood M. A., Wei P., Aytan E., Bozhilov K. N., Balandin A. A., Salguero T. T. (2018). Monoclinic
structures of niobium trisulfide. APL Mater..

[30] Houben L., Enyashin A. N., Feldman Y., Rosentsveig R., Stroppa D. G., Bar-Sadan M. (2012). Diffraction
from Disordered Stacking
Sequences in MoS_2_ and WS_2_ Fullerenes and Nanotubes. J. Phys. Chem. C.

[31] Sourisseau C., Cavagnat R., Fouassier M., Maraval P. (1990). Electronic, vibrational
and resonance Raman spectra of the layered semiconducting compound
NbS_3_. J. Raman Spectrosc..

[32] Childres, I. ; Jauregui, L. A. ; Park, W. ; Cao, H. ; Chen, Y. P. Raman spectroscopy of graphene and related materials. In: Jang, J. I. , Ed., New Developments In Photon And Materials Research; NOVA Science Publishers, Inc., New York 2013, 1, 403–418.

[33] Kozlova M. N., Grayfer E. D., Poltarak P. A., Artemkina S. B., Cherkov A. G., Kibis L. S., Boronin A. I., Fedorov V. E. (2017). Oxidizing
Properties of the Polysulfide Surfaces of Patronite VS_4_ and NbS_3_ Induced by (S_2_)^2–^ Groups: Unusual Formation of Ag_2_S Nanoparticles. Adv. Mater. Interfaces.

[34] Zhou J., Shen Y., Lv F., Zhang W., Lin F., Zhang W., Wang K., Luo H., Wang Q., Yang H. (2022). Ultrathin Metallic NbS_2_ Nanosheets with
Unusual Intercalation Mechanism for Ultra-Stable Potassium-Ion Storage. Adv. Funct. Mater..

[35] Bahl M. K. (1975). ESCA studies
of some niobium compounds. J. Phys. Chem. Solids.

[36] Izawa K., Ida S., Unal U., Yamaguchi T., Kang J.-H., Choy J.-H., Matsumoto Y. (2008). A new approach for the synthesis of layered niobium
sulfide and restacking route of NbS_2_ nanosheet. J. Solid State Chem..

[37] Wang Y., Wu P., Wang Z., Luo M., Zhong F., Ge X., Zhang K., Peng M., Ye Y., Li Q. (2020). Air-Stable Low-Symmetry Narrow-Bandgap 2D Sulfide
Niobium for Polarization
Photodetection. Adv. Mater..

[38] Liang K. S., Cramer S. P., Johnston D. C., Chang C. H., Jacobson A. J., de Neufville J. P., Chianelli R. R. (1980). Amorphous MoS_3_ and WS_3_. J. Non-Cryst. Solids.

[39] Momma K., Izumi F. (2011). VESTA 3 for three-dimensional
visualization of crystal, volumetric
and morphology data. J. Appl. Crystallogr..

[40] Schaffer, B. Digital Micrograph. In Transmission Electron Microscopy: diffraction, Imaging, and Spectrometry; Springer, 2016; pp. 167–196.

[41] Kumagai N., Tanno K., Kumagai N. (1982). Charge—discharge
characteristics
and structural change in various niobium sulfide cathodes for lithium-nonaqueous
secondary batteries. Electrochim. Acta.

[42] Murphy D. W., Trumbore F. A. (1976). The Chemistry of TiS_3_ and NbSe_3_ Cathodes. J. Electrochem. Soc..

[43] Khudorozhko G. F., Asanov I. P., Mischenko A. V., Mazalov L. N., Erenburg S. B., Fedorov V. E., Romanenko A. I. (1988). An X-ray
photoelectron and X-ray
emission study of the electronic structure of Nb trichalcogenides
and their lithium intercalates. J. Solid State
Chem..

[44] Dizhur E., Kostyleva I., Voronovskii A., Zaitzev-Zotov S. (2011). Non-linear
conductivity of NbS3 in pressure induced metal state. Physica Status Solidi C..

[45] Liu C., Zhao G., Zhang L., Yu X., Huang H., Sun K., Zhang N. (2021). A hybrid Mg^2+^/Li^+^ battery based
on high-capacity conversion-type cobalt disulfide cathodes with ultralong
cycle life and high energy density. Chem. Eng.
J..

[46] Tang Y., Tao D., Cao Y., Xu F. (2023). Thermodynamics and kinetics of Mg^2+^/Li^+^ and
Mg^2+^/Na^+^ co-intercalation
into layered titanium disulfide. Phys. Chem.
Chem. Phys..

[47] Yu X., Zhao G., Liu C., Wu C., Huang H., He J., Zhang N. (2021). A MoS_2_ and Graphene Alternately Stacking
van der Waals Heterostructure for Li^+^/Mg^2+^ Co-Intercalation. Adv. Funct. Mater..

[48] Ma Y., Shuai K., Zhou L., Wang J., Wang Q. (2020). Effect of
Mg^2+^ and Mg^2+^/Li^+^ electrolytes on
rechargeable magnesium batteries based on an erythrocyte-like CuS
cathode. Dalton Trans..

[49] Fan X., Tebyetekerwa M., Wu Y., Gaddam R. R., Zhao X. S. (2022). Origin
of Excellent Charge Storage Properties of Defective Tin Disulphide
in Magnesium/Lithium-Ion Hybrid Batteries. Nano-Micro
Lett..

[50] Kravchyk K. V., Widmer R., Erni R., Dubey R. J. C., Krumeich F., Kovalenko M. V., Bodnarchuk M. I. (2019). Copper sulfide nanoparticles as high-performance
cathode materials for Mg-ion batteries. Sci.
Rep.

[51] Duffort V., Sun X., Nazar L. F. (2016). Screening
for positive electrodes for magnesium batteries:
a protocol for studies at elevated temperatures. Chem. Commun..

[52] Chianelli R. R., Dines M. B. (1975). Reaction of butyllithium
with transition metal trichalcogenides. Inorg.
Chem..

[53] O̅nuki Y., Inada R., Tanuma S., Yamanaka S., Kamimura H. (1983). Electrochemical
characteristics of transition-metal trichalcogenides in the secondary
lithium battery. Solid State Ionics.

[54] Mizrahi O., Amir N., Pollak E., Chusid O., Marks V., Gottlieb H., Larush L., Zinigrad E., Aurbach D. (2008). Electrolyte
Solutions with a Wide Electrochemical Window for Rechargeable Magnesium
Batteries. J. Electrochem. Soc..

[55] Tao D., Tang Y., Gui H., Xu F. (2024). Facile Fabrication
of High-Power Molybdenum Diselenide Cathode for Rechargeable Magnesium
Batteries. ACS Sustainable Chem. Eng..

[56] Litwin P. M., Jaszewski S. T., Sarney W. L., Leff A. C., Krylyuk S., Davydov A. V., Ihlefeld J. F., McDonnell S. J. (2023). The growth
of self-intercalated Nb_1+x_Se_2_ by molecular beam
epitaxy: The effect of processing conditions on the structure and
electrical resistivity. J. Vac. Sci. Technol.,
A.

[57] Li W., Wei X., Dong H., Ou Y., Xiao S., Yang Y., Xiao P., Zhang Y. (2020). Colloidal
Synthesis of NbS_2_ Nanosheets: From Large-Area Ultrathin
Nanosheets to Hierarchical
Structures. Front. Chem..

[58] Sugiura C., Kitamura M., Muramatsu S. (1988). Niobium LIII
and LII X-ray absorption-edge
spectra of Nb_2_O_5_ and NH_4_NbF_6_. J. Phys. Chem. Solids.

[59] Fleet M. E., Harmer S. L., Liu X., Nesbitt H. W. (2005). Polarized X-ray
absorption spectroscopy and XPS of TiS_3_: S K- and Ti L-edge
XANES and S and Ti 2p XPS. Surf. Sci..

[60] Saha S., Assat G., Sougrati M. T., Foix D., Li H., Vergnet J., Turi S., Ha Y., Yang W., Cabana J. (2019). Exploring the bottlenecks of anionic redox in Li-rich
layered sulfides. Nat. Energy.

[61] Matsuyama T., Deguchi M., Mitsuhara K., Ohta T., Mori T., Orikasa Y., Uchimoto Y., Kowada Y., Hayashi A., Tatsumisago M. (2016). Structure
analyses using X-ray photoelectron spectroscopy
and X-ray absorption near edge structure for amorphous MS_3_ (M: Ti, Mo) electrodes in all-solid-state lithium batteries. J. Power Sources.

[62] Ohno Y. (1991). Interlayer
interaction in misfit layer compounds MTS_3_ (M = Sn, Pb,La;
T = Ti, Nb). Solid State Commun..

[63] Leube B. T., Robert C., Foix D., Porcheron B., Dedryvère R., Rousse G., Salager E., Cabelguen P.-E., Abakumov A. M., Vezin H. (2021). Activation of anionic
redox in d^0^ transition metal chalcogenides by anion doping. Nat. Commun..

[64] Canadell E., Thieffry C., Mathey Y., Whangbo M. H. (1989). Energy
factors governing
the partial irreversibility of lithium intercalation in layered trichalcogenides
MX_3_ (M = Ti, Zr,Hf; X = S, Se) and the structural changes
in the intercalated species Li_3_MX_3_. Inorg. Chem..

[65] Han X., Meng Q., Wan X., Sun B., Zhang Y., Shen B., Gao J., Ma Y., Zuo P., Lou S. (2021). Intercalation pseudocapacitive electrochemistry
of
Nb-based oxides for fast charging of lithium-ion batteries. Nano Energy.

[66] Liu F., Zhu Z., Chen Y., Meng J., Wang H., Yu R., Hong X., Wu J. (2022). Dense T-Nb_2_O_5_/Carbon Microspheres for Ultrafast-(Dis)­charge and High-Loading Lithium-Ion
Batteries. ACS Appl. Mater. Interfaces.

[67] Cook J. B., Ko J. S., Lin T. C., Robertson D. D., Kim H.-S., Yan Y., Yao Y., Dunn B. S., Tolbert S. H. (2023). Ultrafast Sodium Intercalation Pseudocapacitance in
MoS_2_ Facilitated by Phase Transition Suppression. ACS Appl. Energy Mater..

[68] AfterMath; Pine Research Instrumentation 2023. https://pineresearch.com/shop/kb/knowledge-category/downloads/.

[69] Jing P., Lu H., Yang W., Cao Y. (2020). Interlayer-expanded and binder-free
VS_2_ nanosheets assemblies for enhanced Mg^2+^ and
Li^+^/Mg^2+^ hybrid ion storage. Electrochim. Acta.

[70] Xue X., Chen R., Song X., Tao A., Yan W., Kong W., Jin Z. (2021). Electrochemical Mg^2+^ Displacement
Driven Reversible Copper Extrusion/Intrusion Reactions for High-Rate
Rechargeable Magnesium Batteries. Adv. Funct.
Mater..

