# Supplementary material for: Employing S(-I)/S(-II) Redox Chemistry in Quasi-1D Niobium Trisulfide-Graphene Cathodes for High-Capacity Magnesium–Lithium Hybrid Ion Batteries
Source: ACS Appl Mater Interfaces. 2025 Sep 25;17(40):56040–53. doi: 10.1021/acsami.5c08685 (PMC12516692; doi:10.1021/acsami.5c08685)
Supplement: Supplementary file 1 [file am5c08685_si_001.pdf]

Supporting Information

Employing S(-I)/S(-II) Redox Chemistry in Quasi-1D Niobium Trisulfide-Graphene Cathodes for High-Capacity Magnesium-Lithium Hybrid Ion Batteries

*Pengcheng Jing,[a1] Atsushi Inoishi,[b] Eiichi Kobayashi,[c] Chengcheng Zhao,[a2] Peng Ren,[d1] Isaac Abrahams[d2] and Duncan H. Gregory[a3] **

* Corresponding author

[a1] WestCHEM, School of Chemistry, Joseph Black Building, University of Glasgow, Glasgow, UK, G12 8QQ. E-mail: p.jing.1@research.gla.ac.uk

[b] Institute for Materials Chemistry and Engineering, Kyushu University, Kasuga-koen 6-1, Kasuga, Fukuoka, Japan, 816-8580. E-mail: inoishi@cm.kyushu-u.ac.jp

[c] Kyushu Synchrotron Light Research Center, 8-7 Yayoigaoka, Tosu, Saga, Japan, 841-0005.

E-mail: kobayashi@saga-ls.jp

[a2] WestCHEM, School of Chemistry, Joseph Black Building, University of Glasgow, Glasgow, UK, G12 8QQ. E-mail: 2792248z@student.gla.ac.uk

[d1] Department of Chemistry, Queen Mary University of London, Mile End Road, London, UK, E1 4NS. E-mail: p.ren@qmul.ac.uk

[d2] Department of Chemistry, Queen Mary University of London, Mile End Road, London, UK, E1 4NS. E-mail: i.abrahams@qmul.ac.uk

[a3] WestCHEM, School of Chemistry, Joseph Black Building, University of Glasgow, Glasgow, UK, G12 8QQ. E-mail: Duncan.Gregory@glasgow.ac.uk. Tel: +44-141-330-8128

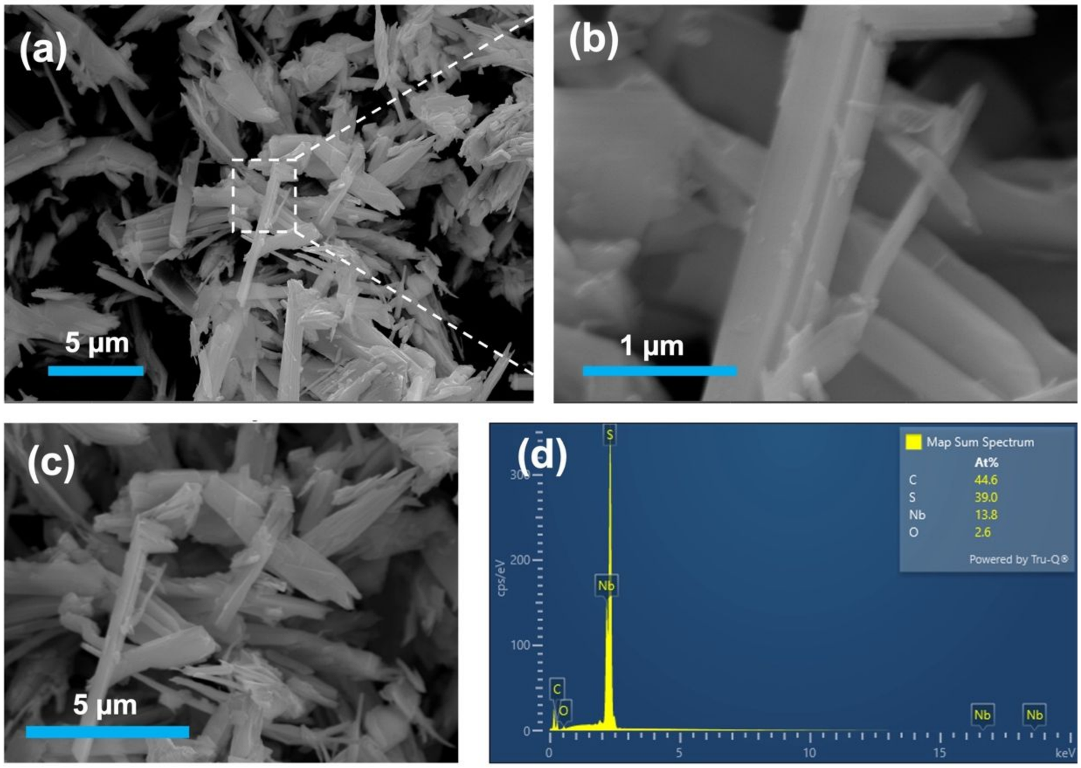

Figure S 1. (a) Low-magnification and (b) corresponding zoomed SEM images of as-synthesised NbS_3 sample. (c) SEM image that was used to acquire (d) EDS spectrum of the whole area.

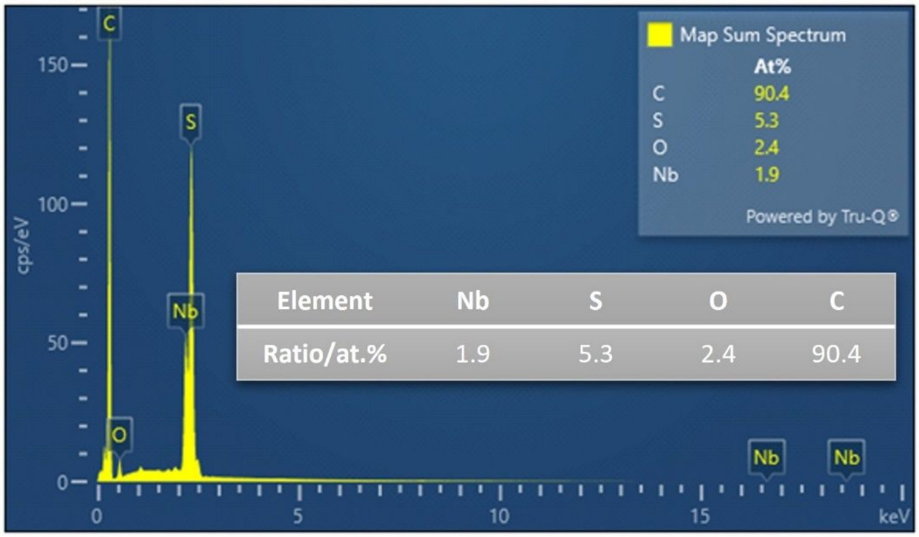

Figure S 2. EDS spectrum of NbS_3G15 taken from the elemental map scan of Figure 2c.

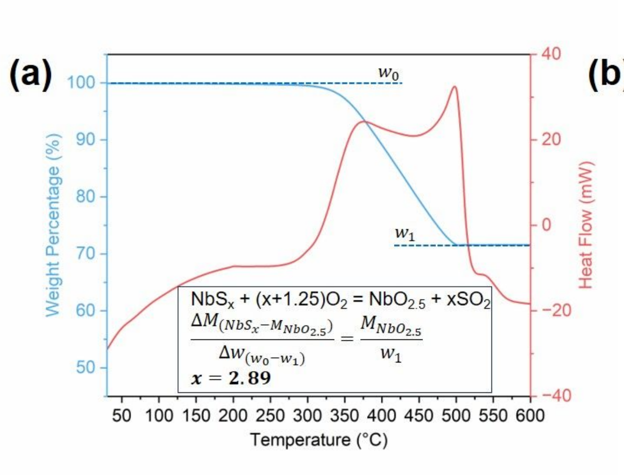

Figure S 3. (a) TG-DSC curves (embedded with the detailed calculations) of the as-synthesised NbS_3 sample and (b) corresponding PXRD pattern of the measurement product (single phase hexagonal Nb_2O_5).

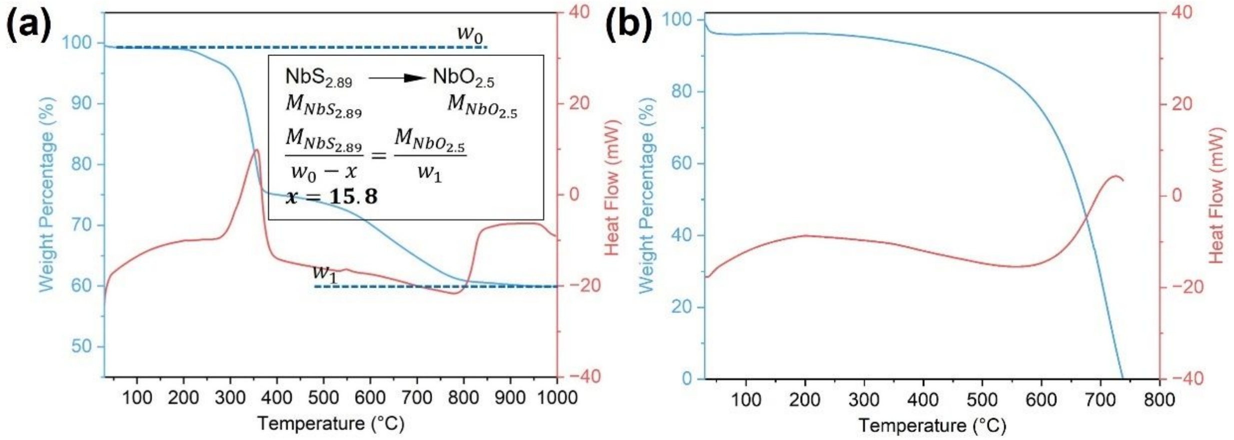

Figure S 4. TG-DSC curves (inserted with the detailed calculations) of (a) the as-prepared NbS_3G15 sample and (b) graphene. The graphene is completely oxidised at *ca.* 737 °C.

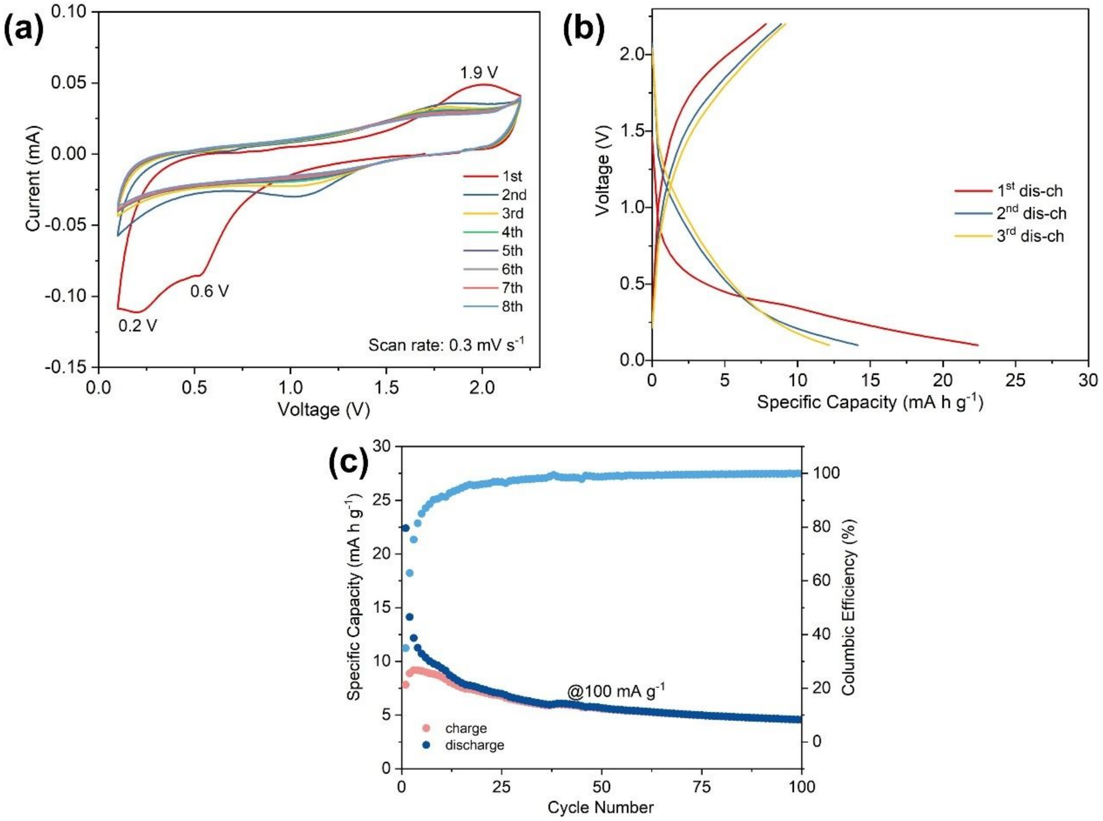

Figure S 5. Electrochemical performance of NbS_3G15 electrodes in MIBs (APC electrolyte): (a) The initial eight CV curves at a scan rate of 0.3 mV s^{-1} between 0.1 V and 2.2 V. (b) The first three (dis)charge curves at a current density of 100 mA g^{-1}. (c) Cycling performance at a current density of 100 mA g^{-1}.

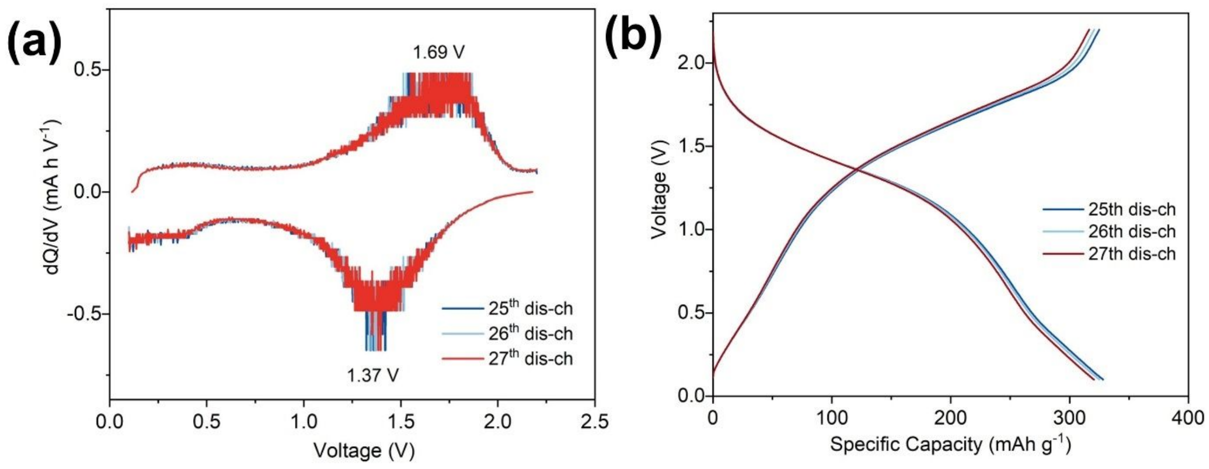

Figure S 6. (a) Differential capacity curves and corresponding (b) 25th - 27th (dis)charge curves of the NbS_3G15 electrode in an MLIB at a current density of 100 mA g^{-1}.

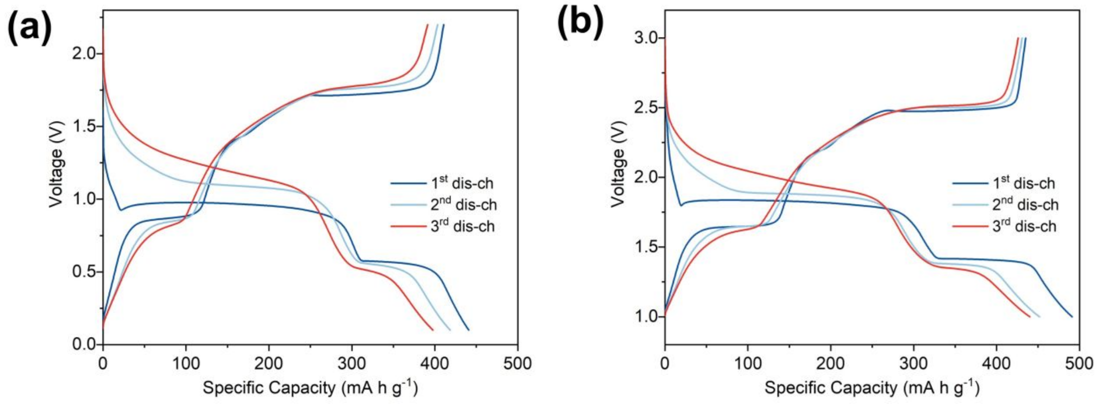

Figure S 7. The initial 3 (dis)charge curves of: (a) the NbS_3 electrode in an MLIB and (b) the NbS_3G15 electrode in an LIB at a current density of 100 mA g^{-1}.

Table S 1. Loadings of NbS_3 in NbS_3G15 and NbS_3 electrodes used in cycling and rate performance

Measurement	NbS_3G15 electrode	NbS_3 electrode
Cycling performance (100 mA g^{-1})	1.14 mg (1.00 mg cm^{-2})	1.54 mg (1.36 mg cm^{-2})
Rate performance	1.52 mg (1.34 mg cm^{-2})	2.17 mg (1.91 mg cm^{-2})
Cycling performance (1000 mA g^{-1})	1.37 mg (1.21 mg cm^{-2})	1.96 mg (1.73 mg cm^{-2})

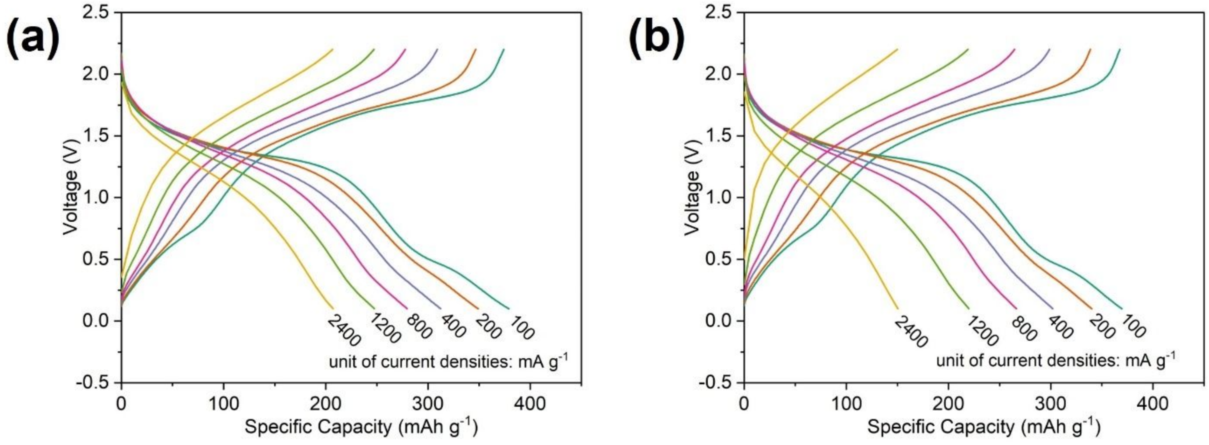

Figure S 8. Selected (dis)charge curves of (a) NbS_3G15 and (b) NbS_3 electrodes in MLIBs at different current densities.

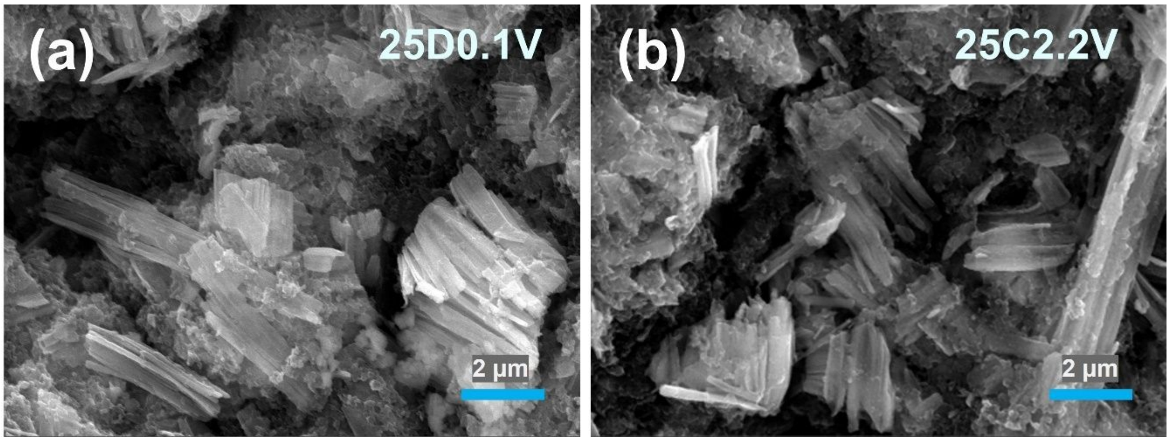

Figure S 9. SEM images of the NbS_3G15 electrodes at (a) 25D0.1V and (b) 25C2.2V states cycled in MLIBs.

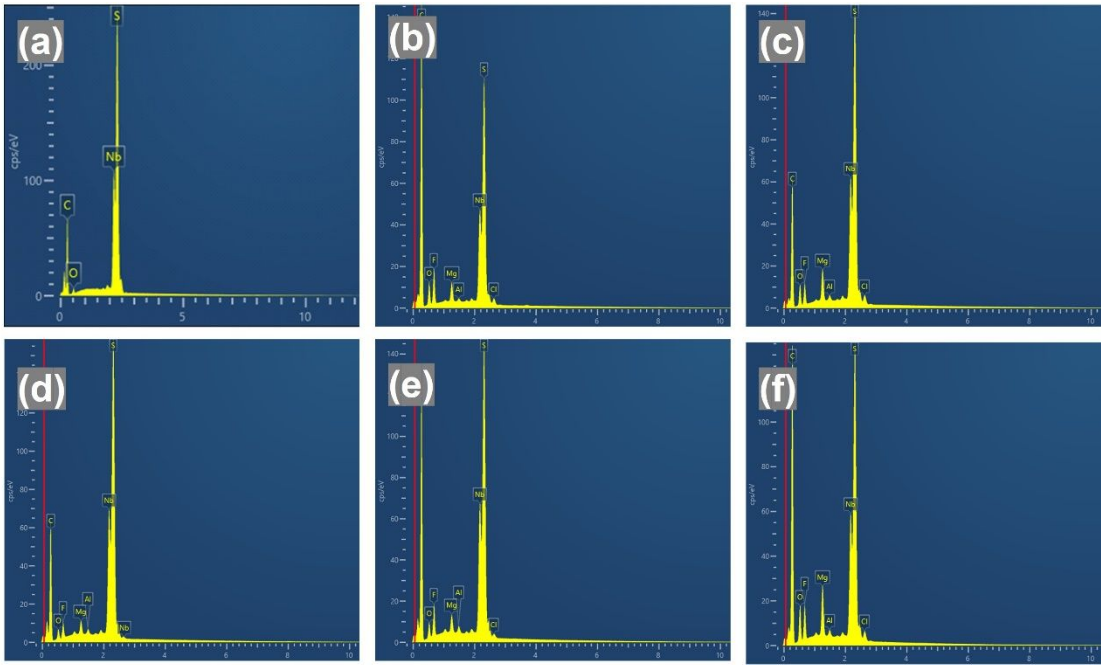

Figure S 10. EDS spectra of an NbS_3G15 electrode: (a) before cycling and after: (b) the 1st discharge to 0.8 V, (c) the 1st discharge to 0.1 V, (d) the 1st charge to 2.2 V, (e) the 2nd discharge to 0.1 V, and (f) the 2nd charge to 2.2 V in an MLIB at a current density of 100 mA g^{-1}.

Table S 2. Atomic composition of an NbS_3G15 electrode at different (dis)charge states as-obtained from Figure S10.

	As detected/ at.%					Atomic ratio		
	Nb/%	S/%	Mg/%	Cl/%	Al/%	Mg/Nb	Cl/Mg	Cl/Al
Pristine	26.1	73.9	0.0	0.0	0.0	0.00	-	-
1D0.8 V	23.4	66.2	6.5	2.6	1.3	0.28	0.40	2.00
1D0.1 V	23.3	65.1	7.5	2.7	1.4	0.32	0.36	2.00
1C2.2 V	25.2	70.1	3.4	-	1.4	0.14	-	-
2D0.1 V	22.1	62.1	11.6	3.2	1.1	0.52	0.27	3.00
2C2.2 V	25.5	67.3	4.1	1.0	2.0	0.16	0.25	0.50

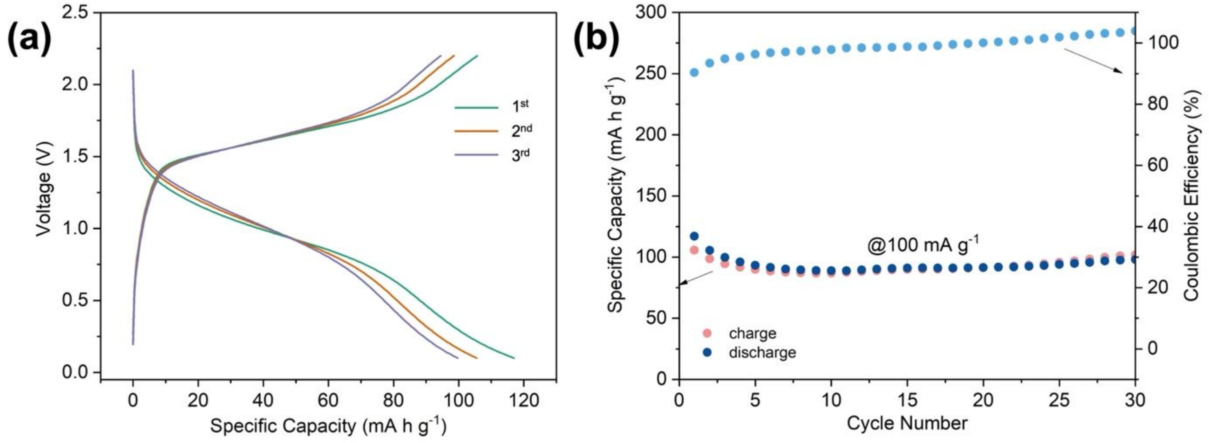

Figure S 11. (a) Discharge-charge curves and (b) cycling performance of the activated NbS_3G15 electrode in MLIB at a current density of 100 mA g^{-1}.

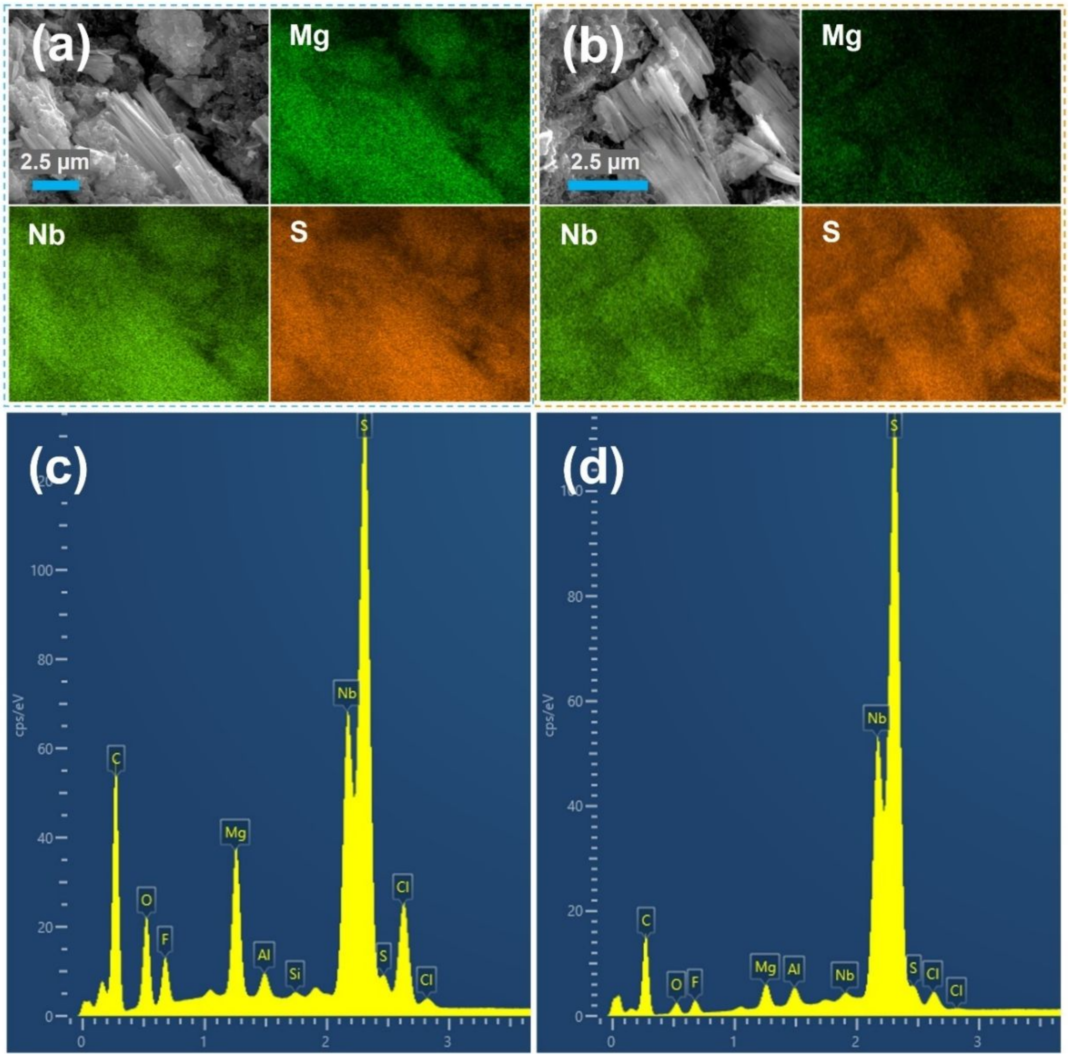

Figure S 12. SEM images and corresponding EDS maps (Mg, Nb, and S) and spectra of the NbS_3G15 electrodes at (a, c) the 25th discharged and (b, d) 25th charged states.

Table S 3. Atomic composition of an NbS_3G15 electrode at the 25th discharged and charged states as-obtained from Figure S11e, f.

As detected/ at.%	Atomic ratio

	Nb	S	Mg	Cl	Al	Mg/Nb	Cl/Mg	Cl/Al
25th D 0.1 V	21.7	55.0	15.8	13.3	2.5	0.7	0.8	5.3
25th C 2.2 V	24.6	67.3	2.8	3.3	1.9	0.1	1.2	1.8

Table S 4. Values of fitted XPS peaks for various oxidation states of respective elements in the NbS_3G15 electrode from Figures 4g, h.

**State**	**Binding energy (eV) of constitute elements at various oxidation states**					
	Oxidation State	Nb $3d_{5/2}$	Nb $d_{3/2}$	Oxidation State	S $2p_{3/2}$	S $2p_{1/2}$
Before cycle	Nb^{4+} sulfide	203.9	206.6	S^{-}	162.4	163.6
	Nb^{5+} oxide	207.1	209.8	S^{2-}	161.2	162.3
1D0.08V	Nb^{4+} sulfide	204.2	206.8	S^{-}	-	-
	Nb^{4+} oxide	205.1	207.9	S^{2-}	160.8	162.1
	Nb^{5+} oxide	207.0	209.6	S^{0}	163.3	164.5
1D0.1V	Nb^{4+} sulfide	204.3	206.9	S^{-}	-	-
	Nb^{3+} sulfide	203.4	206.1	S^{2-}	160.9	162.1
	Nb^{4+} oxide	205.1	207.9			
	Nb^{5+} oxide	207.1	209.8			
1C2.2V	Nb^{4+} sulfide	204.2	206.9	S^{-}	162.4	163.5
	Nb^{5+} oxide	207.1	209.7	S^{2-}	161	162.3
2D0.1V	Nb^{4+} sulfide	204.2	206.9	S^{-}	-	-
	Nb^{3+} sulfide	203.4	206.1	S^{2-}	160.9	162.1
	Nb^{4+} oxide	205.1	207.8			

	Nb^{5+} oxide	207.1	209.6			
2C2.2V	Nb^{4+} sulfide	204.2	206.8	S^{-}	162.4	163.5
	Nb^{5+} oxide	207.0	209.6	S^{2-}	161.2	162.3

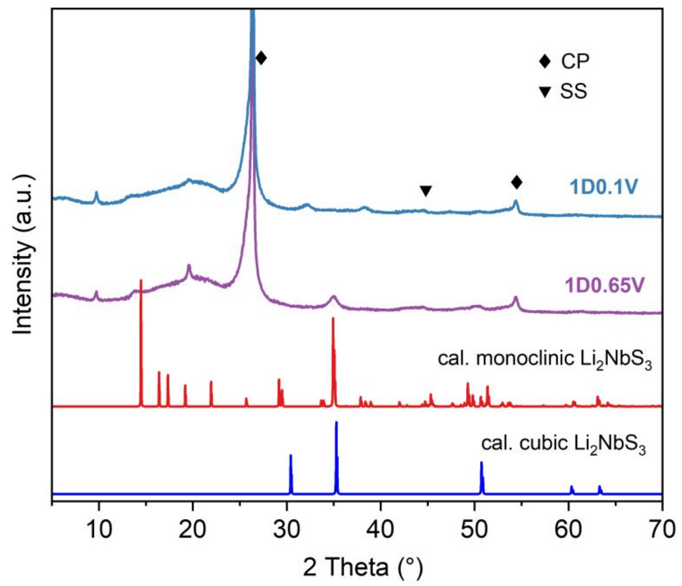

Figure S 13. Selected *operando* PXRD patterns of the NbS_3G15 electrode discharged to 1D0.65V (phase 1) and 1D0.1V (phase 2) and the calculated PXRD of monoclinic and cubic Li_2NbS_3 phases. The reflections of carbon paper (CP; rhombus) and stainless steel (SS; triangle) have been highlighted.

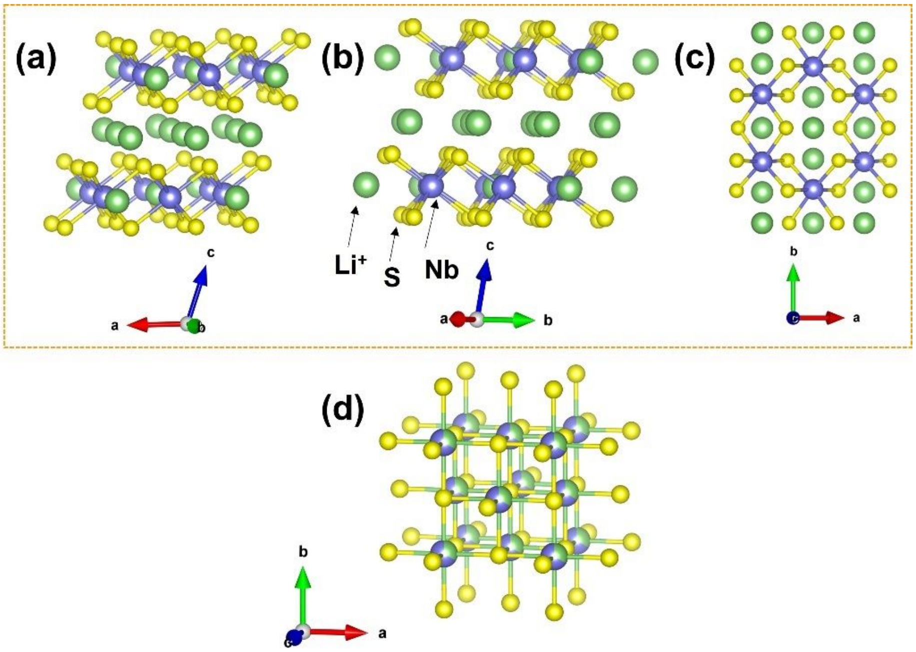

Figure S 14. Crystal structure of monoclinic Li_2NbS_3 projected along near (a) *ac* plane, (b) near *bc* plane, and (c) *ab* plane. Green, purple, and yellow balls represent Li^+, Nb^{4+}, and S^{2-} ions, respectively. (d) Crystal structure of cubic Li_2NbS_3 projected along near *ab* plane. The occupancies of Li^+ and Nb^{4+} are 0.33 and 0.67, respectively.

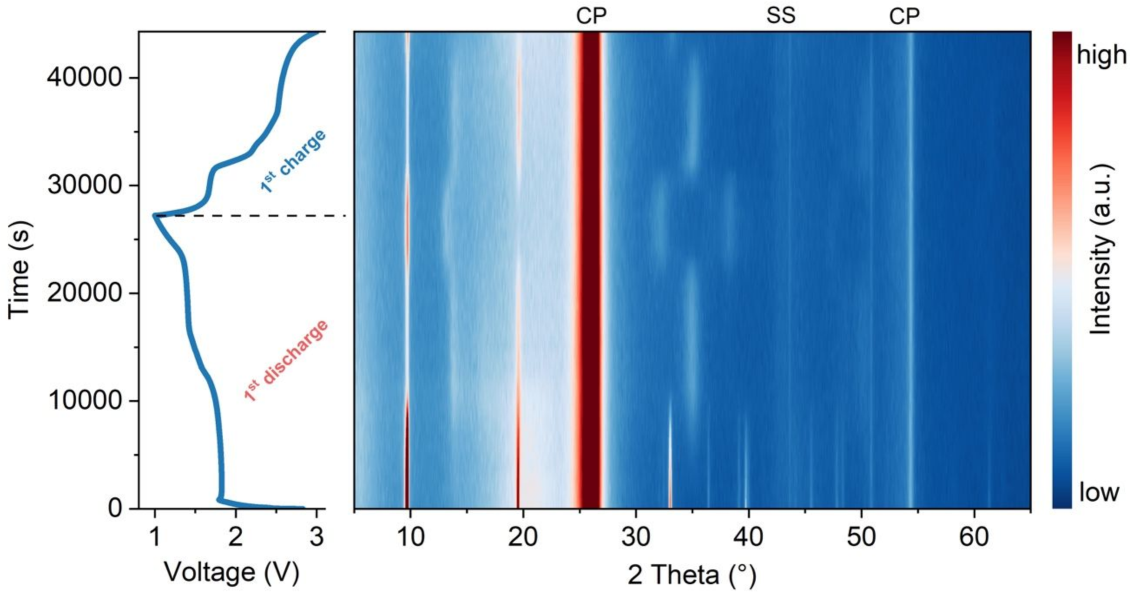

Figure S 15. (a) The first discharge-charge curve and (b) corresponding contour plot of the operando PXRD patterns of the NbS_3G15 electrode in lithium battery at a current density of 100 mA g^{-1}.

The CV peak current can be expressed by a power law relationship as shown in Equation S1:[1]

$$i = av^b \quad \text{Equation S1}$$

where i is the current (mA), v is the scan rate (mV s^{-1}), and a and b are adjustable constants. For pure diffusion-mediated processes, i is proportional to $v^{1/2}$ (b = 0.5), whereas for capacitative processes, i is proportional to v (b = 1). In cases where both mechanisms are involved, b typically falls between 0.5 to 1.0. A linear fit of log(i) versus log(v) (Equation S2) provides insights into the nature of charge storage mechanisms:

$$log(i) = log(a) + blog(v) \quad \text{Equation S2}$$

The current contributions from surface-confined capacitive processes (k_1) and bulk diffusion-controlled processes (k_2) at any voltage can be resolved using Equation S3 by performing a linear fit of $i/v^{1/2}$ against $v^{1/2}$.[2]

$$i = k_1 v + k_2 v^{1/2} \text{ or } \frac{i}{v^{1/2}} = k_1 v^{1/2} + k_2 \qquad \text{Equation S3}$$

The diffusion coefficient (D) can be calculated based on Fick's law using Equation S4:[3]

$$D = \frac{4}{\pi\tau}\left(\frac{m_B V_M}{M_B S}\right)^2 \left(\frac{\Delta Es}{\Delta E\tau}\right)^2 \qquad \text{Equation S4}$$

Here, τ is the constant current pulse duration (s); m_B, V_M, M_B, and S are the mass (g) of the active material, molar volume (cm^3 mol^{-1}), molar mass (g mol^{-1}), and contact area of the electrode with the electrolyte (cm^{-2}), respectively. ΔE_T represents the potential change during the current pulse, excluding IR drops (vertical potential jumps at the onset or termination of the current pulse, attributable to ohmic and charge transfer resistances).[4, 5] ΔE_s denotes the potential difference between equilibrium states before and after the current pulse. Both ΔE_T and ΔE_s can be obtained from GITT curves (Figure 7d).

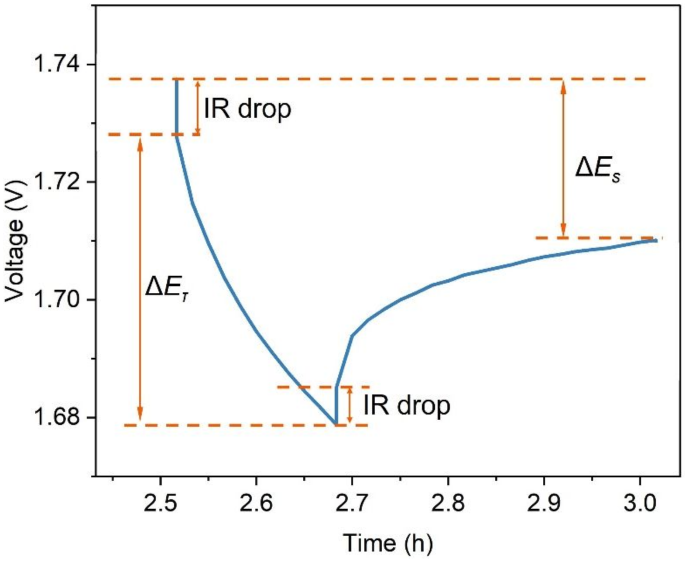

Figure S 16. One pair of current pulse and relaxation taken from the GITT curve of the NbS_3G15 electrode shown in Figure 7d revealing ΔE_T, ΔE_s, and *IR* drop, respectively.

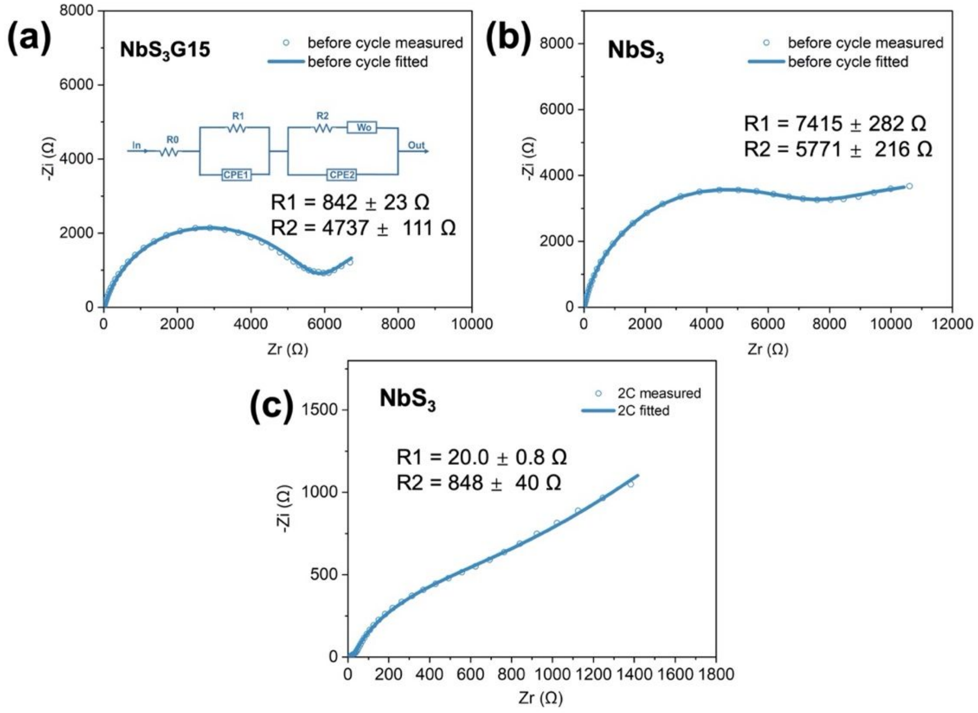

Figure S 17. Measured (circles) and fitted (solid line) EIS spectra of (a) the (-)Mg|LiAPC|NbS_3G15(+) cell (in the uncycled state), and (-)Mg|LiAPC|NbS_3(+) cell before and after the 2nd cycle. The modelled equivalent circuit is embedded as an inset in the graph.

Table S 5. Specific values of parameters in the obtained equivalent circuit model for the three (-)Mg|LiA PC|NbS_3G15(+) cells at before cycling, post 2nd charged and 25th charged states.

		Q_1(s□ Ω^{-1})	Q_2(s□ Ω^{-1})	R_0(Ω)	R_1(Ω)	R_2(Ω)	W_0(Ω $s^{-0.5}$)
Before cycle	value	5.63E-05	2.21E-05	8.29	841.95	4737.08	609.56
	error	4.92E-13	9.87E-07	0.22	22.77	111.45	22.24
2nd charge	value	2.49E-05	2.30E-04	11.00	9.89	238.13	280.64
	error	3.64E-13	7.35E-06	0.31	0.63	12.07	19.70

25th charge	value	9.78E-05	7.27E-04	13.66	11.40	56.35	59.30
	error	4.25E-12	7.26E-05	1.70	1.71	8.61	6.59

References

(1) Ren, W.; Zhang, H.; Guan, C.; Cheng, C. Ultrathin MoS_2 Nanosheets@Metal Organic Framework-Derived N-Doped Carbon Nanowall Arrays as Sodium Ion Battery Anode with Superior Cycling Life and Rate Capability. *Advanced Functional Materials* **2017**, *27* (32), 1702116. DOI: https://doi.org/10.1002/adfm.201702116.

(2) Wang, J.; Polleux, J.; Lim, J.; Dunn, B. Pseudocapacitive Contributions to Electrochemical Energy Storage in TiO_2 (Anatase) Nanoparticles. *The Journal of Physical Chemistry C* **2007**, *111* (40), 14925-14931. DOI: https://doi.org/10.1021/jp074464w.

(3) Wen, C. J.; Boukamp, B. A.; Huggins, R. A.; Weppner, W. Thermodynamic and Mass Transport Properties of "LiAl" *Journal of The Electrochemical Society* **1979**, *126* (12), 2258. DOI: https://doi.org/10.1149/1.2128939.

(4) Knehr, K. W.; Biswas, S.; Steingart, D. A. Quantification of the Voltage Losses in the Minimal Architecture Zinc-Bromine Battery Using GITT and EIS. *Journal of The Electrochemical Society* **2017**, *164* (13), A3101. DOI: https://doi.org/10.1149/2.0821713jes.

(5) Jing, P.; Stevenson, S.; Lu, H.; Ren, P.; Abrahams, I.; Gregory, D. H. Pillared Vanadium Molybdenum Disulfide Nanosheets: Toward High-Performance Cathodes for Magnesium-Ion Batteries. *ACS Applied Materials & Interfaces* **2023**, *15* (44), 51036-51049. DOI: https://doi.org/10.1021/acsami.3c10287.
